# Evidence that alternative transcriptional initiation is largely nonadaptive

**DOI:** 10.1371/journal.pbio.3000197

**Published:** 2019-03-18

**Authors:** Chuan Xu, Joong-Ki Park, Jianzhi Zhang

**Affiliations:** 1 Department of Ecology and Evolutionary Biology, University of Michigan, Ann Arbor, Michigan, United States of America; 2 Division of EcoScience, Ewha Womans University, Seoul, Republic of Korea; University of Bath, UNITED KINGDOM

## Abstract

Alternative transcriptional initiation (ATI) refers to the frequent observation that one gene has multiple transcription start sites (TSSs). Although this phenomenon is thought to be adaptive, the specific advantage is rarely known. Here, we propose that each gene has one optimal TSS and that ATI arises primarily from imprecise transcriptional initiation that could be deleterious. This error hypothesis predicts that (i) the TSS diversity of a gene reduces with its expression level; (ii) the fractional use of the major TSS increases, but that of each minor TSS decreases, with the gene expression level; and (iii) *cis*-elements for major TSSs are selectively constrained, while those for minor TSSs are not. By contrast, the adaptive hypothesis does not make these predictions a priori. Our analysis of human and mouse transcriptomes confirms each of the three predictions. These and other findings strongly suggest that ATI predominantly results from molecular errors, requiring a major revision of our understanding of the precision and regulation of transcription.

## Abstract 2

The transcription of a gene may start from one of several transcription start sites, a phenomenon known as alternative transcriptional initiation. Contrary to common belief, this study shows that variation of the transcription start site of a given gene is nonadaptive and is largely attributable to transcriptional initiation error that is typically deleterious.

## Author summary

Multiple surveys of transcriptional initiation showed that mammalian genes typically have multiple transcription start sites such that transcription is initiated from any one of these sites. Many researchers believe that this phenomenon is adaptive because it allows production of multiple transcripts, from the same gene, that potentially vary in function or post-transcriptional regulation. Nevertheless, it is also possible that each gene has only one optimal transcription start site and that alternative transcriptional initiation arises primarily from molecular errors that are slightly deleterious. This error hypothesis makes a series of predictions about the amount of transcription start site diversity per gene, relative uses of the various start sites of a gene, among-tissue and across-species differences in start site usage, and the evolutionary conservation of *cis*-regulatory elements of various start sites, all of which are verified in our analyses of genome-wide transcription start site data from the human and mouse. These findings strongly suggest that alternative transcriptional initiation largely reflects molecular errors instead of molecular adaptations and require a rethink of the precision and regulation of transcription.

## Introduction

The transcription start site (TSS) is the first nucleotide transcribed in a run of transcription, while the surrounding genomic region of the TSS is often referred to as the core promoter [1]. Owing to the strong association between TSSs and core promoters, these terms are sometimes used interchangeably [1]. Under an appropriate external signal, the core promoter forms a transcription preinitiation complex with a number of accessory proteins including RNA polymerase and transcription factors to initiate transcription [1–5]. Needless to say, regulation of transcriptional initiation is a crucial step in the control of gene expression [6, 7].

The transcription of a gene may start from one of several TSSs, a phenomenon known as alternative transcriptional initiation (ATI); the different core promoters used are referred to as alternative promoters [8, 9]. It has been reported that ATI occurs to most eukaryotic protein-coding genes [6, 7, 10–12]. For example, over 50% of all human genes have alternative promoters [13], and on average, a human gene has four TSSs [7]. ATI allows the production from the same gene of transcripts differing in the 5′ untranslated region (5′ UTR) or even the protein-coding region. ATI-dependent variations of the 5′ UTR may impact the translational efficiency of the transcript [14]. One example is the human runt-related transcription factor 1 gene *RUNX1*, which can be transcribed from two different TSSs; the mRNA produced from the distal TSS mediates cap-dependent translation, whereas that from the proximal TSS contains a functional internal ribosome entry site (IRES) and mediates cap-independent translation [15]. ATI-dependent variations of the coding region may affect protein function. For instance, human *LEF1*, encoding lymphoid enhancer binding factor 1 that regulates the transcription of Wingless/Integrated (Wnt)/β-catenin genes, produces two different protein isoforms by using alternative TSSs; the longer isoform recruits β-catenin to Wnt target genes, whereas the shorter isoform cannot interact with β-catenin and instead suppresses the Wnt regulation of target genes [16].

A number of case studies showed that the TSS choice may vary among tissues [17], across developmental stages [18, 19], or during cell differentiation [20] and that aberrations in the TSS choice can lead to various diseases [21–23]. Such findings led to the adaptive hypothesis that ATI is a widely used, regulated mechanism to expand the transcriptome and/or proteome diversity [7–9, 24–26].

Nevertheless, alternative TSSs with verified benefits account for only a tiny fraction of all known TSSs, while the vast majority of TSSs have unknown functions. More than 90,000 TSSs are annotated for approximately 20,000 human protein-coding genes in ENSEMBL genome reference consortium human build 37 (GRCh37). Recent surveys using high-throughput sequencing methods such as deep cap analysis gene expression (deepCAGE) [27] showed that human TSSs are much more abundant than what has been annotated [7]. Are most TSSs of a gene functionally distinct, and is ATI generally adaptive? While this possibility exits, here we propose and test an alternative, nonadaptive hypothesis that is at least as reasonable as the adaptive hypothesis. Specifically, we propose that there is only one optimal TSS per gene and that other TSSs arise from errors in transcriptional initiation that are mostly slightly deleterious. This hypothesis is based on the consideration that transcriptional initiation has a limited fidelity [28], and harmful ATI may not be fully suppressed by natural selection if the harm is sufficiently small or if the cost of fully suppressing harmful ATI is even larger than the benefit from suppressing it [29]. The error hypothesis makes a series of distinct predictions about patterns of ATI that are not expected a priori under the adaptive hypothesis. By analyzing high-throughput mRNA 5′-end sequencing data from multiple cell lines and tissues of humans and mice, we provide unequivocal evidence for the error hypothesis. This finding echoes a number of recent discoveries that mechanisms thought to adaptively increase transcriptomic and/or proteomic diversities, such as alternative splicing, alternative polyadenylation, and RNA editing, are all largely manifestations of molecular errors.

## Results

### TSS diversity decreases with gene expression level

Under the error hypothesis of ATI, TSS diversity arises from imprecise transcriptional initiation that could be harmful for several reasons. First, the transcript generated may miss certain regulatory sequences for translation, influencing the protein production. Second, the transcript may lack part of the coding sequence, leading to a reduction, loss, or alteration of the protein function. Third, the transcript may have altered upstream open read frames (uORFs), interfering with normal protein synthesis. Because some of these harms, such as the toxicity of the dysfunctional proteins produced or energy waste owing to the synthesis of functionless proteins, increase with the number of protein molecules synthesized, the overall deleterious effect of imprecise transcriptional initiation of a gene is expected to rise (but not necessarily linearly) with its mRNA concentration [30]. Consequently, natural selection against the transcriptional initiation error intensifies with the rise of the gene expression level. As a result, the error rate and TSS diversity should decline with the rise of the gene expression level. By contrast, this trend is not predicted a priori by the adaptive hypothesis, under which the TSS diversity of a gene depends on the specific function and regulation of the gene. To distinguish between the error hypothesis and the adaptive hypothesis of ATI, we analyzed the TSSs identified by 5′-end sequencing (CAGE-seq and TSS-seq; see Materials and methods) of numerous cell lines and tissues from humans and mice (**S1 Table)**.

**Fig 1:**
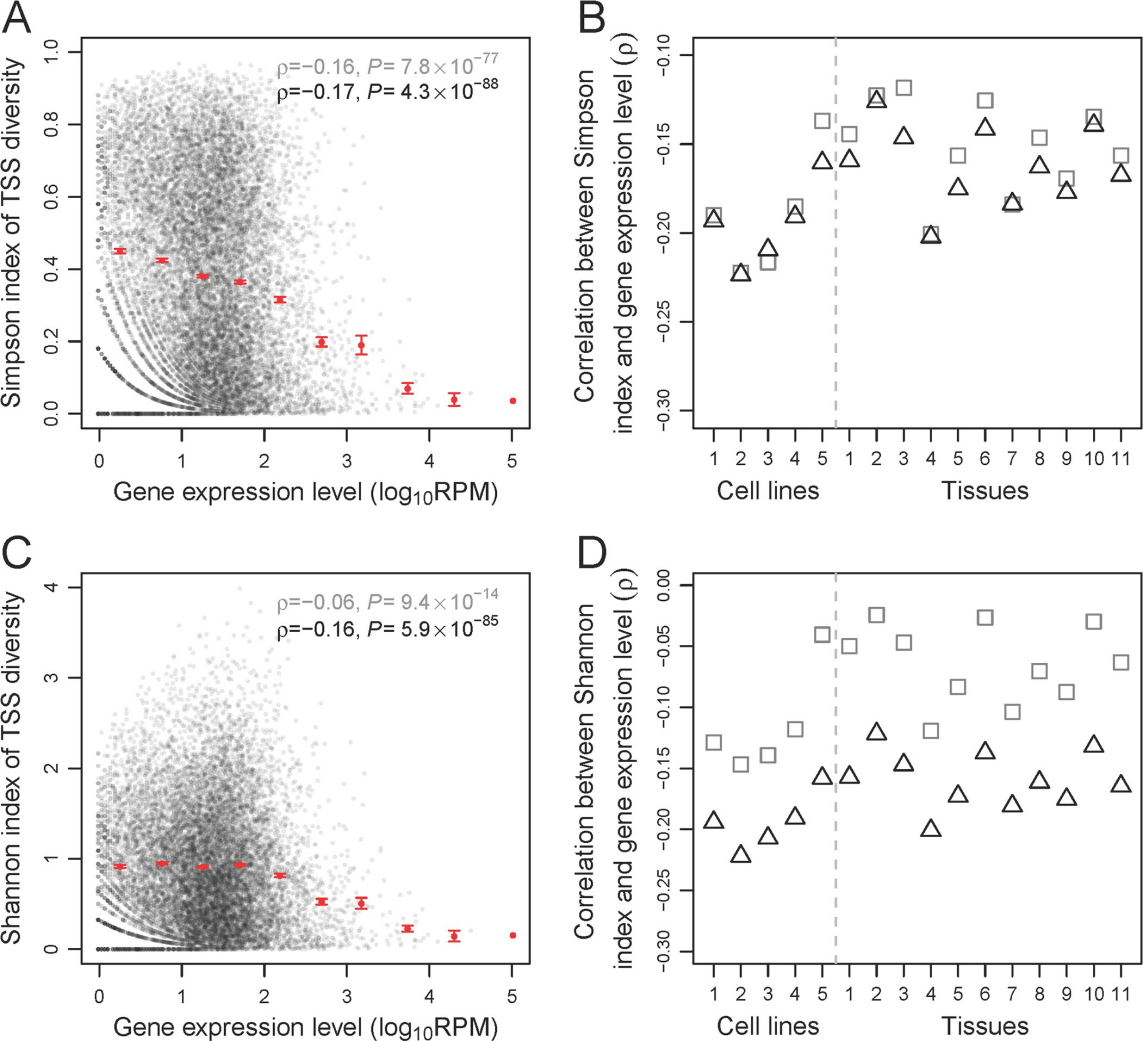
The TSS diversity of a gene generally decreases with the gene expression level. (A) The Simpson index of TSS diversity of a gene in the human universal sample declines with the expression level of the gene in the sample. (B) Spearman's correlations between gene expression level and Simpson index of TSS diversity in each of five human cell lines and 11 human tissue samples examined. (C) The Shannon index of TSS diversity of a gene in the human universal sample declines with the expression level of the gene in the sample. (D) Spearman's correlations between gene expression level and Shannon index of TSS diversity in each human cell line and tissue sample examined. In (A) and (C), each black dot represents a gene. Spearman's rank correlation coefficient (ρ) and associated *P*-value are presented for the original unbinned data (gray) and down-sampled data (black), respectively. Each red dot shows the mean X-value and mean Y-value of the genes in each of 10 equal-interval bins (i.e., all bins have the same log_10_RPM interval), while the error bars show standard errors (error bar is absent when a bin contains only one gene). In (B) and (D), gray squares and black triangles show the correlations on the basis of the original unbinned data and down-sampled data, respectively. *P* < 5 × 10^−3^ for all correlations. Sample IDs listed on the *x*-axis refer to those in S1 Table. Data are available at https://github.com/ZhixuanXu/Nonadaptive-alternative-TSSs. ID, identifier; RPM, reads mapped to the gene per million reads; TSS, transcription start site.

Following our recent study of alternative polyadenylation [31], we used the Simpson index [32] and Shannon index [33] to quantify the TSS diversity of each protein-coding gene in each sample (see Materials and methods). Both indices are commonly used in biodiversity research and tend to rise with the number of TSSs in a gene as well as the evenness of the relative uses of these TSSs, but the Simpson index gives more weight to the frequently used TSSs than does the Shannon index. Because CAGE-seq is regarded as the best among many different 5′-end RNA sequencing (RNA-seq) methods [34], our analyses mainly used TSSs identified by the CAGE-seq high-throughput data of functional annotation of the mouse/mammalian genome project (FANTOM) [7]. Let us define a gene by the DNA segment between its 5′-most TSS annotated and its 3′-most end among all annotated transcripts. We concentrated on so-called permissive TSSs (see Materials and methods), which are located within a gene or within 500 bp upstream of the 5′-most end of the gene. Because TSS diversity is poorly estimated when the number of sequencing reads mapped to a gene is too small, only genes with at least 10 reads in a sample were analyzed. By counting the CAGE-seq reads corresponding to each TSS in a gene, we calculated the gene’s TSS diversity. The gene expression level was measured by the number of CAGE-seq reads mapped to the gene per million reads (RPM) mapped in the entire sample. We started by analyzing the human universal sample, which is a mixture of 10 cell lines originating from different human tissues [35]. Consistent with the prediction of the error hypothesis, the rank correlation (ρ) between the expression level of a gene and its Simpson index of TSS diversity is significantly negative, and this negative correlation is apparent across the entire expression range (**Fig 1A**). The magnitude of ρ appears small, likely because estimates of gene expression levels and TSS diversities have relatively large sampling errors, especially for lowly expressed genes. When the genes are grouped into 100 bins with the same expression range size or the same gene number, the rank correlation between the mean expression level and mean TSS diversity across the 100 bins becomes –0.96 and –0.84, respectively, suggesting that gene expression level is a major determinant of TSS diversity. To rule out the possibility that the observed negative correlation is an artifact of our analysis, we performed a computer simulation. Briefly, we randomly generated genes whose expression level and relative TSS usages respectively follow the gene-expression–level distribution and relative TSS usage distribution of the real genes. We analyzed the simulated genes as if they were the actual data but found a weak, positive correlation between expression level and TSS diversity (**S2 Table**), confirming that the negative correlation observed in the actual data is not an artifact of our statistical analysis.

To investigate the robustness of the above results, we performed several additional analyses. First, because annotated TSSs are generally considered genuine, we focused on TSSs that are within 500 bp from each annotated TSS in a gene [7], and the obtained result (**S1A Fig**) is highly similar to that from all TSSs. Second, using so-called robust TSSs (see Materials and methods) should reduce false-positive TSSs. The obtained result, however, remains qualitatively unchanged (**S1B Fig**). Third, because some genuine TSSs identified by CAGE-seq may be far upstream from the most upstream TSS currently annotated for a gene [36], we included TSSs that are located within 1, 5, or 10 kb upstream of the most upstream TSS annotated for the gene. But the results were qualitatively unchanged (**S1C–S1E Fig**). Fourth, while CAGE-seq has been demonstrated to be as reliable as the canonical mRNA sequencing (RNA-seq) in measuring gene expression levels [37], it is valuable to examine whether our observation holds when gene expression levels are measured by RNA-seq. To this end, we replaced the gene expression level in **Fig 1A** with that obtained from canonical RNA-seq of the same sample [38]. As expected, the result is similar (**S1F Fig**).

Because sequencing depth and the precision of TSS survey for a gene rise with its expression level, it is possible that the correlation in **Fig 1A** originates from unequal TSS surveys of genes of different expression levels. To eliminate this potential bias, we down-sampled our data by randomly picking 10 CAGE-seq reads per gene for all genes with at least 10 reads and then re-estimated the Simpson index. The correlation (ρ′) between the gene expression level and the re-estimated Simpson index remains negative (**Fig 1B**; see **S2 Table** for the simulation result). Similar patterns were observed in other human cell lines and tissues (**Figs 1B and S1**). To examine the robustness of our results from the down-sampled data, we down-sampled CAGE-seq reads to as few as 5 and as many as 80 reads per gene from genes with at least that many reads and found our results to remain qualitatively unchanged (**S2 Fig**). Using the Shannon index to measure TSS diversity similarly yielded a negative correlation between gene expression level and TSS diversity, as shown in **Fig 1C** for the human universal sample, **Fig 1D** for the other human cell lines and tissues, and **S2 Fig** for different levels of down-sampling.

To further examine the robustness of the above CAGE-seq–based results, we analyzed two different and independent 5′-end RNA-seq data: TSS-seq and 5′ global run-on sequencing (GRO-cap) (see Materials and methods). We examined 42 human cell line/tissue data (**S1 Table**) generated by TSS-seq and observed significant, negative correlations between TSS diversity and gene expression level in the vast majority of these samples (**S3A Fig**). In addition, we analyzed the GRO-cap data from the human cell line K562, allowing direct comparison of the result with our CAGE-seq–based result from the same cell line. The GRO-cap data show a significantly negative correlation between TSS diversity and gene expression level (ρ = –0.16 for both the original and down-sampled data; **S3B Fig**), similar to that from the CAGE-seq data (ρ = –0.19 for both the original and down-sampled data; the fourth cell line in **Fig 1B**).

To minimize the influences of potential confounding factors in the above analyses, we compared between human paralogous genes of different expression levels because paralogous genes are similar in gene structure, DNA sequence, regulation, and function [39]. Consistent with the error hypothesis, Simpson and Shannon indices tend to be lower for the relatively highly expressed gene than the relatively lowly expressed one in a pair of paralogous genes, and this trend generally holds after CAGE-seq reads are down-sampled to 10 per gene **(S4 Fig**).

To examine whether the negative correlation between gene expression level and TSS diversity observed in humans also holds in other mammals, we analyzed the CAGE-seq–based TSS data of 11 mouse tissues (**S1 Table)** available at FANTOM5 phase1.3 [7]. The mouse results (**S5 Fig**) resembled the human results, indicating that the negative correlation is not a human-specific phenomenon.

### Usages of all but the major TSSs decline with the gene expression level

**Fig 2:**
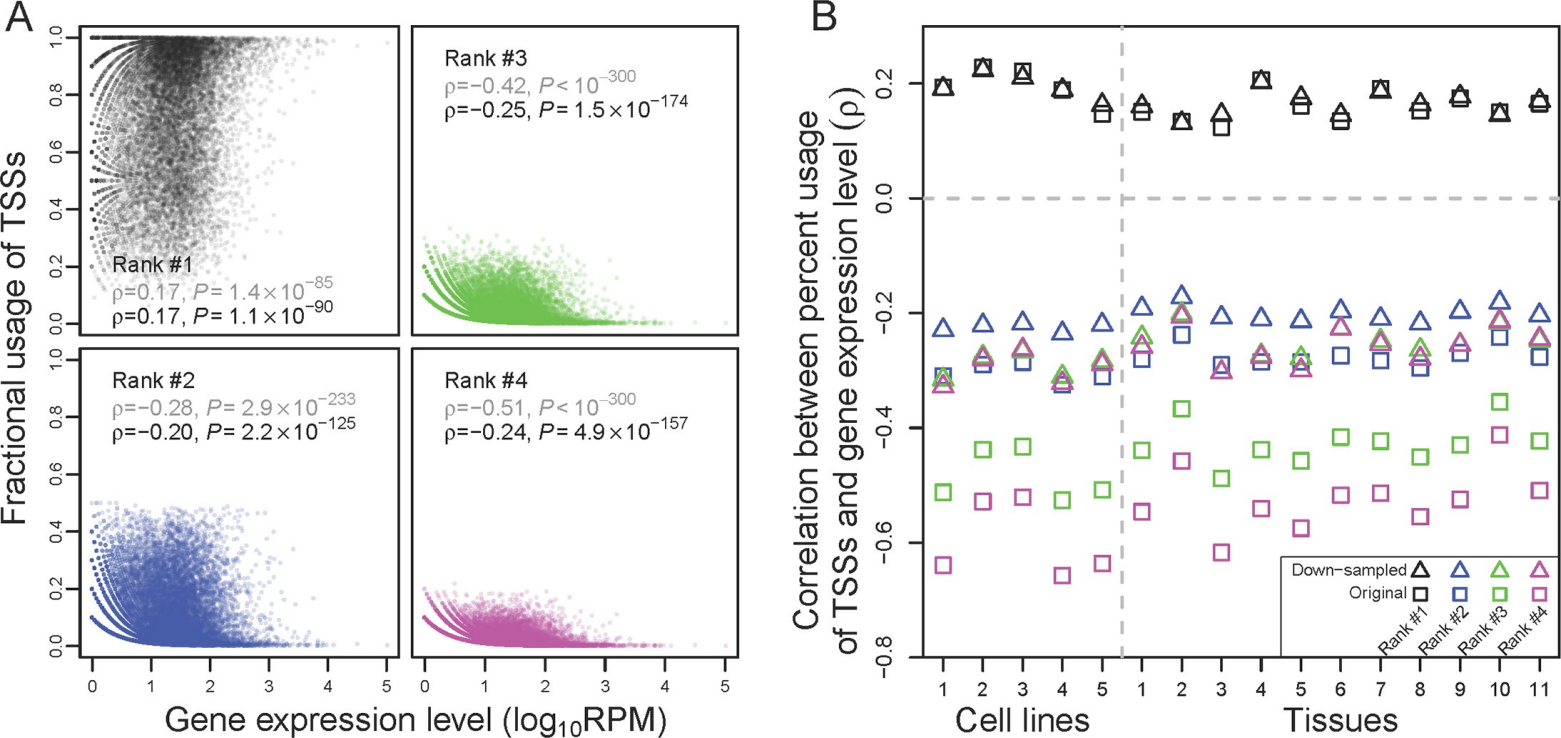
Increased fractional use of the most frequently used TSS of a gene and decreased fractional use of each other TSS when gene expression level rises. (A) Spearman's correlation (ρ) between the expression level of a gene and the fractional uses of its TSSs in the human universal sample. TSSs are ranked on the basis of their fractional uses in the sample concerned, with rank #1 being the most frequently used one (major TSS). Each dot represents a gene. Gray and black ρ and *P* are based on the original and down-sampled data, respectively. (B) Spearman's rank correlation between the expression level of a gene and the fractional uses of its TSSs in each human cell line or tissue sample examined. *P* < 10^−39^ in all cases. Squares and triangles show the correlations on the basis of the original and down-sampled data, respectively. In both panels, the correlation for TSSs with a particular rank is calculated using the genes that have at least that particular number of TSSs. Sample IDs listed on the *x*-axis of (B) refer to those in S1 Table. Data are available at https://github.com/ZhixuanXu/Nonadaptive-alternative-TSSs. ID, identifier; RPM, reads mapped to the gene per million reads; TSS, transcription start site.

The above analyses suggest that many TSSs of a gene are suboptimal such that the overall TSS diversity declines with the gene expression level as a result of natural selection against suboptimal TSSs, but how many of the TSSs are suboptimal and which ones are suboptimal are unclear. To address these questions, we ranked all TSSs of a gene by their fractional usages. The fractional usage of a TSS is the number of CAGE-seq reads mapped to the TSS divided by the total number of reads mapped to all TSSs of the gene. For a given gene, the TSS with the highest fractional usage (i.e., ranked #1) is referred to as the major TSS, while all others are referred to as minor TSSs. Intuitively, the major TSS should be a preferred TSS. Because natural selection against transcriptional initiation error intensifies with the gene expression level, the fractional usage of each preferred TSS should increase while that of each unpreferred TSS should reduce as the expression level increases. We first tested this prediction in the human universal sample. Again, we considered only genes with at least 10 CAGE-seq reads to ensure a certain level of accuracy in TSS usage estimation. Indeed, the fractional usage of the major TSS in a gene increases with its expression level (upper-left plot in **Fig 2A**). By contrast, each minor TSS examined shows the opposite trend, suggesting that none of them is preferred. For example, among all genes with at least two TSSs, the fractional usage of the second most frequently used TSS in a gene decreases with gene expression level (lower-left plot in **Fig 2A**). A similar negative correlation is observed for the third most frequently used TSSs among genes with at least three TSSs (upper-right plot in **Fig 2A**) and for the fourth most frequently used TSSs among genes with at least four TSSs (lower-right plot in **Fig 2A**). These trends remain unchanged when only TSSs within 500 bp around each annotated TSS of each gene are considered (**S6A Fig**); when only robust TSSs of each gene are considered (**S6B Fig**); when TSSs located within 1, 5, or 10 kb upstream of the most upstream TSS annotated for a gene are considered (**S6C–S6E Fig**); or when gene expression levels are measured by RNA-seq (**S6F Fig**). We also observed a negative correlation when the analysis in **Fig 2A** is extended to the fifth, sixth, seventh, and eighth most frequently used TSSs among genes with at least five, six, seven, and eight TSSs, respectively. We further verified the results in **Fig 2A** by down-sampling the original data to 10 CAGE-seq reads per gene and reranking TSSs using the down-sampled data (**Fig 2B**). Computer simulations confirmed that these trends are not statistical artifacts (**S2 Table**). CAGE-seq data from other human tissues and cell lines (**Figs 2B and S6**) as well as TSS-seq data from multiple tissues and cell lines (**S7 Fig**) show similar patterns. We also verified that the statistical trends in **Fig 2**generally hold even when we limited the analysis to the common set of genes with at least four TSSs (**S8A Fig**). Analysis of mouse tissues yielded similar results (**S8B** and **S8C Fig**). These observations strongly suggest that, for most genes, in any tissue or cell line, only the major TSS is preferred while all other TSSs are unpreferred.

We also validated the above human results using paralogous genes, which should be more comparable as mentioned. For the human universal sample, in 56% of the 1,962 pairs of paralogous genes analyzed, the major TSS is used more often in the relatively highly expressed paralog than in the relatively lowly expressed one, significantly more than the random expectation of 50% (**S9A Fig**). By contrast, for the second, third, and fourth most frequently used TSSs, respectively, significantly smaller than 50% of gene pairs show higher fractional usages in the relatively highly expressed gene than in the relatively lowly expressed one (**S9A Fig**). Other tissues and cell lines show similar patterns (**S9B Fig**). These trends generally hold in down-sampled data (**S9B Fig**).

### Among-cell–type variations in TSS usage support the error hypothesis

**Fig 3:**
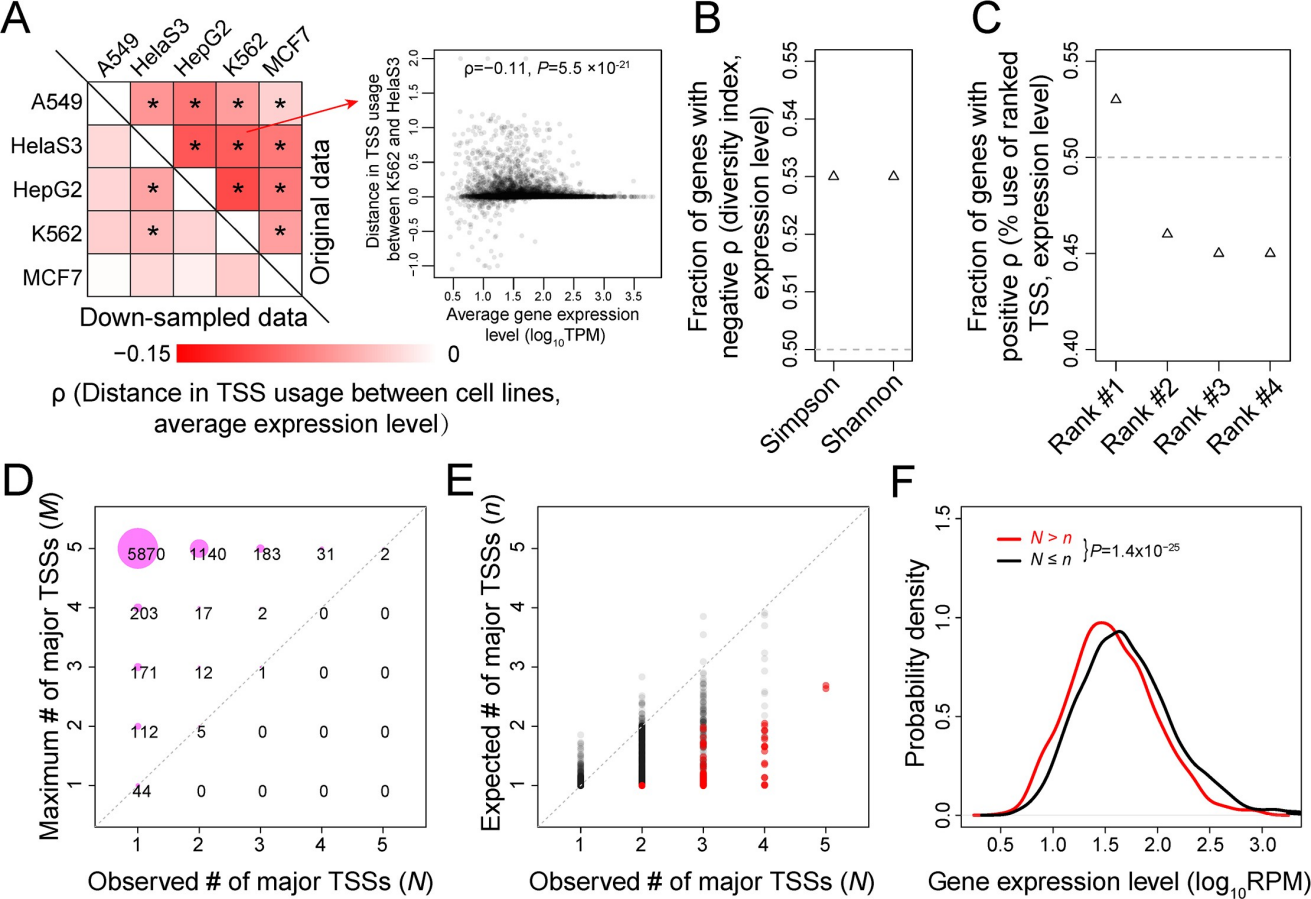
Variation in TSS usage among five human cell lines. (A) Spearman's correlations between the mean expression level of a gene in two cell lines and the between-cell–line distance in TSS usage. Above and below the diagonal are results obtained from the original and down-sampled data, respectively. All correlations are negative; those significant at *P* = 0.05 are indicated by an asterisk. The scatter plot for the comparison between K562 and HeLa S3 is presented as an example. (B) Fraction of genes with a negative among-cell–line Spearman's correlation between the Simpson or Shannon index of TSS diversity and expression level. (C) Fraction of genes with a positive among-cell–line Spearman's correlation between the gene expression level and fractional use of a ranked TSS. In (B) and (C), results are based on down-sampled data and *P* < 10^−4^ in all cases (binomial test). (D) The maximum number (*M*) of different major TSSs that a gene can have (given its observed TSSs) in the five human cell lines is greater than the observed number (*N*) of different major sites for almost all genes with *M* ≥ 2. The area of a circle is proportional to the indicated number of genes in the circle. (E) Only in a minority of human genes is the number (*N*) of observed major TSSs significantly greater than that (*n*) expected under no differential use of TSSs among five human cell lines. Each dot represents a gene, with red dots denote genes whose *N* exceeds *n* significantly (*Q* < 0.05). No gene has a significantly lower *N* than *n* (*Q* < 0.05). (F) The probability densities of expression level for genes with larger *N* than *n* (not necessarily significantly; red) and the rest of the genes (black). In this panel, *N* and *n* have been re-estimated using down-sampled data to equalize the sampling error among genes. Data are available at https://github.com/ZhixuanXu/Nonadaptive-alternative-TSSs. HepG2, human liver cancer cell line Hep G2; MCF7, human breast cancer cell line MCF-7; RPM, reads mapped to the gene per million reads; TSS, transcription start site.

The above analyses of ATI in each tissue or cell line support our hypothesis that there is only one optimal TSS in each tissue or cell line and that all other TSSs are suboptimal. Now, we compare TSS usage among cell types. Because a tissue is usually composed of many different cell types, comparison among tissues is less precise than that among cell lines. We thus analyzed the CAGE-seq data of five human cell lines. Under the error hypothesis of ATI, any difference in TSS usage between cell types is due to the stochastic nature of transcriptional initiation error. Hence, the hypothesis predicts that this difference decreases as the expression level of the gene rises, because of the reduced transcriptional initiation errors of more highly expressed genes. By contrast, no such prediction is made a priori by the adaptive hypothesis because the difference in ATI between cell types would depend on the specific cell types and genes. To this end, we measured the distance in the fractional uses of TSSs of a gene between two cell types (see Materials and methods) and then correlated the distance with the gene's mean expression level in the two cell types compared. We found that in all 10 pairs of cell lines compared, the correlation is significantly negative (**Fig 3A**), supporting the error hypothesis. To avoid the influence of different sequencing depths of different genes, we sampled 10 CAGE-seq reads per gene from each cell line for all genes that have at least 10 reads in each of the five cell lines and confirmed that all correlations remain negative despite some that become statistically insignificant (**Fig 3A**).

The error hypothesis further predicts that, when the expression level of a gene varies among cell types, the TSS diversity of the gene in a cell type decreases with the rise of its expression level in the cell type. To verify this prediction, we calculated for each gene the correlation between its expression level and TSS diversity across the five human cell lines. We focused on down-sampled data to guard against the influences of unequal sequencing depths of a gene across cell lines. Indeed, significantly more genes exhibit negative correlations than expected by chance regardless of whether we used the Simpson or Shannon index of TSS diversity (**Fig 3B**).

The error hypothesis further predicts a negative correlation across cell types between the expression level of a gene and the fractional use of each minor TSS. To verify this prediction, for each gene, we defined the major and minor TSSs in each cell line separately and then computed the across-cell–line rank correlation between the expression level of a gene in a cell line and the fractional use of a TSS of a certain rank in the cell line (based on down-sampled data). Indeed, 53% of genes show a positive correlation for the TSSs of rank #1, significantly more than the random expectation of 50%, whereas only 45–46% of genes show a positive correlation for each TSS of ranks #2, #3, and #4, significantly lower than the random expectation (**Fig 3C**).

Although our evidence so far suggests that, for most genes, each cell type has only one preferred TSS, it remains possible that the optimal TSS varies among cell types such that the ATI variation among cell types is adaptive. To assess this possibility, we first repeated the analysis in **Fig 3C** by defining the global major and minor TSSs for each gene using the combined CAGE-seq reads from all five cell lines. Interestingly, the patterns observed are similar to those in **Fig 3C**. For example, 53% of genes show a positive correlation between the fractional usage of the TSS of global rank #1 in a cell line and the expression level of the gene in the cell line, significantly more than the random expectation (**S10 Fig**). But for all global minor TSSs examined, only 46–47% of genes show a positive correlation, significantly less than the random expectation (**S10 Fig**). Along with the observations in **Fig 3C**, these results suggest that only a small fraction of genes may have different optimal TSSs in different cell types. To estimate this fraction, we first counted the number of different major TSSs observed in each gene across the five cell lines (*N*; **Fig 3D**) because if all five cell lines share the same major TSS, it is most likely that they all share the same optimal TSS. We found that, of 7,793 genes examined, 6,400 (or 82.1%) have the same major site in all five cell lines (i.e., *N* = 1). We also examined the maximum possible number of different major TSSs in the five cell lines (*M*) for each gene, which would be the smaller of 5 and the total number of TSSs observed in the five cell lines for the gene (**Fig 3D**). When *M* ≥ 2, 99.9% of genes show *N* < *M*, suggesting that cell-type–specific optimization of transcription start sites is far less than what ATI could potentially offer, consistent with the hypothesis that among-cell–type variations in ATI are largely nonadaptive.

Even when different cell lines show different major TSSs, the optimal TSS could still be the same in these cell lines because the observation could be due to sampling error caused by limited sequencing depths. To examine this possibility, for each gene, we randomly shuffled its CAGE-seq reads among the five cell lines without altering the number of reads in each cell line and then used the shuffled data to count the number of different major TSSs in the five cell lines. We repeated this process 10,000 times and estimated the mean number of major TSSs for the gene in the shuffled data (*n*) (**Fig 3E**) and the fraction of times (*f*) when the number of major TSSs observed in the shuffled data equals to or exceeds that in the actual data. Here, *f* is an estimate of the one-tailed *P*-value in testing the null hypothesis that all cell lines share the same major TSS. We converted the *P*-values to *Q*-values to control for multiple testing and found that 343 genes have *Q* < 0.05 (red dots in **Fig 3E**). Thus, approximately 4.4% of genes examined appear to have at least two different optimal TSSs in the five cell lines.

The above analysis assumed that the major TSS of a gene in a cell line is the optimal TSS in the cell line, but this may not always be the case because using the optimal TSS more than any other TSS in every cell type for every gene could be difficult because of the limited power of transcription start regulation. Thus, it is possible that, even after the exclusion of sampling error, the observed major TSS is still not the optimal site for some genes in some cell types. This kind of high usage of suboptimal sites should have a fitness cost that rises with the gene expression level. Consequently, this phenomenon should have lower occurrences in more highly expressed genes as a result of natural selection. To this end, for each gene, we sampled 10 CAGE-seq reads from each cell line and re-estimated *N* and *n* as was done for the original data. We then divided all genes into two groups: those with *N* > *n* (regardless of statistical significance of this inequality) and the rest. The expression level is significantly lower for the former group of 1,582 genes than the latter group of 6,211 genes (**Fig 3F**). This observation supports the idea that a sizable proportion of genes with *N* > *n* do not necessarily have different optimal sites in different cell types; rather, they cannot use the single optimal TSS in all cell types as the major TSS. In other words, the fraction of genes with evidence for adaptive differential ATI among cell types is lower than the above estimate of 4.4%. Notwithstanding, statistical power for detecting differential optimal TSSs among cell types rises with the sequencing depth, and the potential for the existence of at least two different optimal TSSs among cell types increases with the number of cell types examined. Hence, the above value of 4.4% is tentative, and this question should be revisited in the future with a larger and better data set.

### Between-species ATI differences are consistent with the error hypothesis

**Fig 4:**
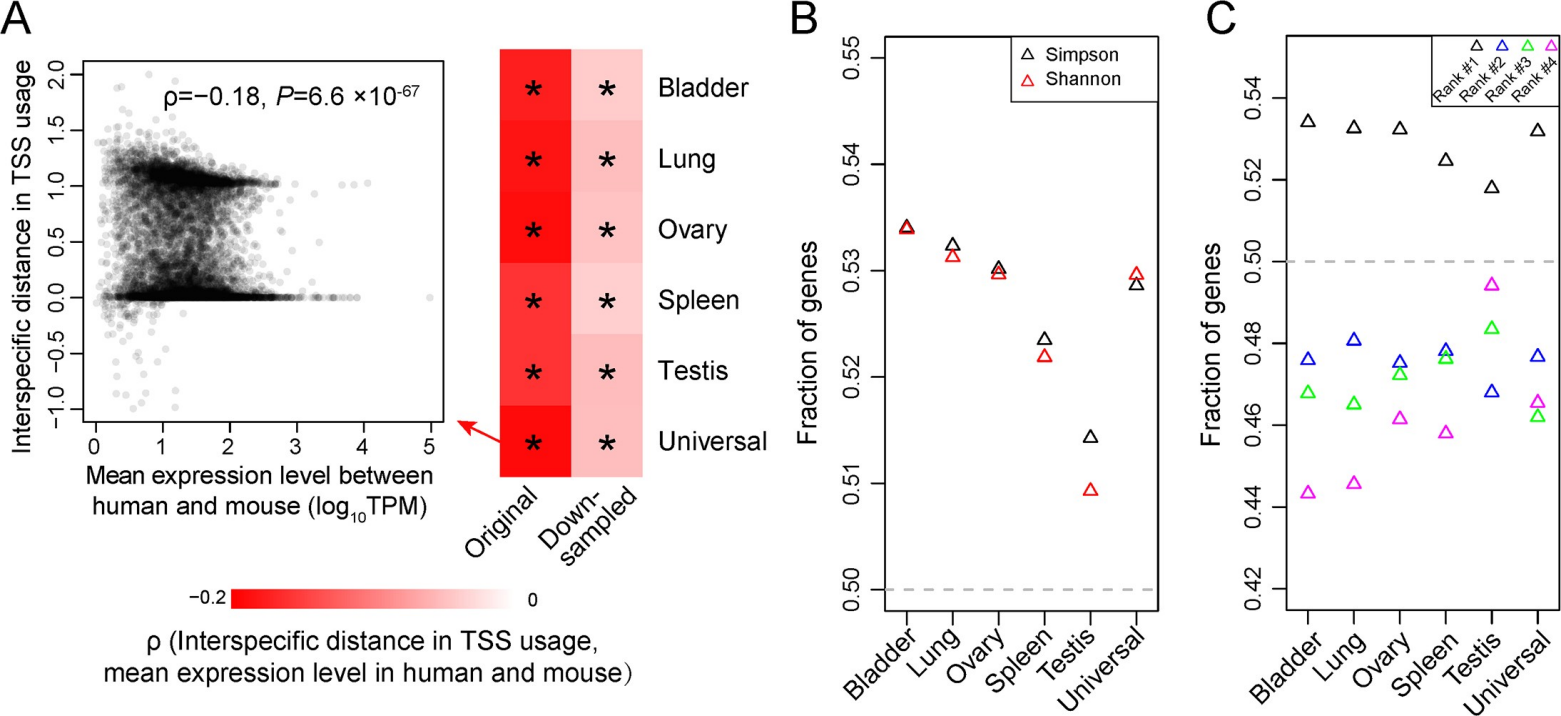
TSS usages of human–mouse orthologous genes in each of six tissue samples. (A) Spearman's correlations between the mean expression level of a gene in the two species and its interspecific distance in TSS usage. All correlations are negative; those significant at *P* = 0.05 are indicated by an asterisk. The scatter plot of the human–mouse comparison of the universal sample is presented as an example. (B) The fraction of genes for which the Simpson or Shannon index of TSS diversity is lower in the species where the gene expression level is higher. All fractions significantly exceed the random expectation of 50% (*P* < 0.05) except for those in the testis. (C) Fraction of genes for which the percent usage of the TSS of a particular rank is higher in the species where the gene expression level is higher. All fractions deviate significantly from the random expectation of 50% (*P* < 0.05). In (B) and (C), down-sampled data are used. Data are available at https://github.com/ZhixuanXu/Nonadaptive-alternative-TSSs. TPM, transcripts per million; TSS, transcription start site.

The error hypothesis predicts that the interspecific difference in fractional uses of various TSSs of a gene should decrease as the average expression level of the gene in the two species rises because highly expressed genes have reduced TSS diversity in each species. By contrast, no such trend is predicted a priori by the adaptive hypothesis. To distinguish between the two hypotheses, we measured the distance in the fractional uses of TSSs of one-to-one orthologous genes between human and mouse in the same tissue (see Materials and methods). In each tissue, we correlated between this distance and the mean expression level of the gene in the two species across genes. We considered only genes with at least 10 CAGE-seq reads in the pair of human and mouse tissues concerned to ensure relatively accurate measures of TSS usage. In support of the error hypothesis, a significantly negative correlation is found in each of the six tissues examined (**Fig 4A**), and these trends hold in down-sampled data (**Fig 4A**).

Because a gene may have different expression levels between human and mouse in a given tissue, the error hypothesis predicts that its TSS diversity should be lower in the species in which its expression level is higher. Indeed, using down-sampled data, we observed this trend in all six tissues **(Fig 4B**). Furthermore, for most genes, the fractional use of the major TSS identified in a species increases with the expression level of the gene in the species (**Fig 4C**). For each minor TSS, the opposite is true (**Fig 4C**).

### Natural selection on the *cis*-elements of core promoters

**Fig 5:**
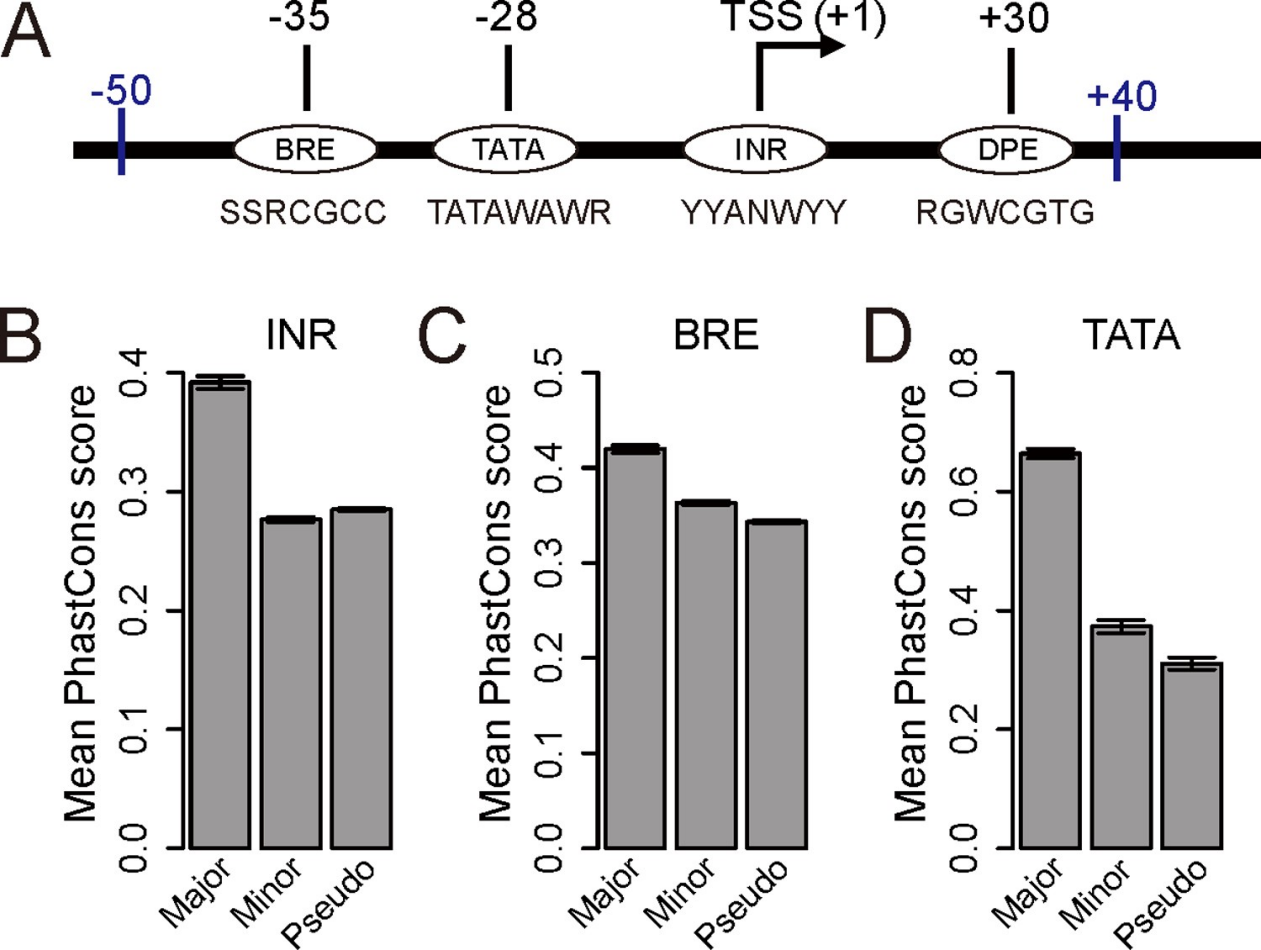
Evolutionary conservations of *cis*-elements of human core promoters. (A) The typical structure of a core promoter and consensus sequences of *cis*-elements. The most likely positions in nts relative to the TSS (+1) are given for core promoter *cis*-elements. (B–D) Mean PhastCons scores of *cis*-elements of global major TSSs, *cis*-elements of global minor TSSs, and pseudoelements for INR (B), BRE (C), and TATA (D). In (B)–(D), the mean PhastCons score is significantly different (*P* < 0.05, Mann–Whitney *U* test) between any pair of the three bins. Error bars show the standard error. Degenerate nucleotide symbols used are as follows. N: A, G, C, or T; H: A, T, or C; W: A or T; R: A or G; Y: C or T; M: A or C; K: G or T; S: G or C. Data are available at https://github.com/ZhixuanXu/Nonadaptive-alternative-TSSs. BRE, TFIIB recognition element; DPE, downstream promoter element; INR, initiator; nt, nucleotide; TATA, TATA box; TSS, transcription start site.

That a nucleotide site is used as a TSS is because of the existence of a nearby core promoter, which is commonly composed of four well characterized *cis*-elements: the TATA box, the initiator (INR), the TFIIB recognition element (BRE), and the downstream promoter element (DPE) [2]. These *cis*-elements jointly determine the activity of the core promoter and hence the use of the corresponding TSS (**Fig 5A**). Our finding that, for most human genes, the major TSS is likely optimal while all minor TSSs are likely suboptimal predicts that the *cis*-elements corresponding to the major TSS should be evolutionarily conserved, while those corresponding to minor TSSs should not be conserved and may even be selected against. To test this prediction, we merged all CAGE-seq reads of 15 independent cell lines and tissues of humans to determine the global major and minor TSSs of each gene. For comparison, we identified the *cis*-elements from the corresponding segment of the complementary strand of DNA; they are referred to as pseudoelements because they are not expected to be functional. Because some *cis*-elements of a core promoter may overlap with the coding, intron, or UTR of a transcript corresponding to another promoter, we considered only the *cis*-elements in regions that have never been annotated as coding sequence (CDS), intron, or 3′ UTR in any transcript, as well as the pseudoelements in the corresponding regions. Because the DPE is located in the transcribed region, we could not examine its evolutionary constraint that is purely due to the DPE function. Therefore, we focused on the other three *cis*-elements. We used PhastCons scores across 46 mammals as a measure of evolutionary conservation [40]. We found that the PhastCons scores are substantially greater for the *cis*-elements of major TSSs than for the *cis*-elements of minor TSSs or pseudoelements (**Fig 5B–5D**). As predicted, the PhastCons scores are similar between the *cis*-elements of minor TSSs and pseudoelements (**Fig 5B–5D**). Specifically, INRs exhibit a slightly but significantly lower conservation in minor TSSs than in pseudoelements (**Fig 5B**), probably reflecting natural selection against the existence of minor TSSs and/or attributable to weak purifying selection on pseudoelements associated with functional antisense transcription. The conservations of BREs and TATA boxes are slightly but significantly higher for minor TSSs than for pseudoelements (**Fig 5C and 5D**). Because some minor TSSs are located downstream of the major TSS such that their conservations could be due to their positions in the 5′ UTR of the primary transcript instead of their promoter activities, we further analyzed only the minor TSSs upstream of the major TSS. Now INRs (**S11A Fig**) and TATA boxes (**S11C Fig**) both show substantially lower conservations in minor TSSs than pseudoelements, whereas BREs show slightly higher conservations than pseudoelements (**S11B Fig**). These results generally support the prediction of the error hypothesis.

## Discussion

The prevalence of ATI of human and mouse genes has been known for years, and the prevailing view is that ATI is mostly adaptive, although evidence supporting this view exists in only a small number of genes [8, 9, 24, 25]. In this study, we proposed that ATI is largely a manifestation of deleterious transcriptional initiation error. By analyzing multiple 5′-end sequencing data and *cis*-elements corresponding to TSSs, we provided strong evidence for the above error hypothesis in mammals. While most of our evidence was based on the tissues and cell lines analyzed (**S1 Table**), the analysis of the evolutionary conservation of *cis*-elements was based on genome sequences, and hence our conclusion that ATI is largely nonadaptive is not restricted to the specific tissues and cell lines examined. Assuming that deleterious ATI has not been selectively purged in genes of the lowest expressions but has been completely removed in those of the highest expressions, we can treat ATI of lowly expressed genes as the total ATI (*T*) and ATI of highly expressed genes as nondeleterious ATI (*ND*). Thus, the fraction of ATI that is deleterious is (*T*-*ND*)/*T* = 1-*ND*/*T*. We used the Simpson index of TSS diversity to measure the amount of ATI because both the number of TSSs and their relative usages are considered. In the human universal sample depicted in Fig 1A, the 20 most weakly expressed genes have a mean Simpson index of 0.530 (*T*), while the 20 most highly expressed genes have a mean Simpson index of 0.069 (*ND*). Hence, the fraction of deleterious ATI is 1 –*ND*/*T* = 1–0.069/0.53 = 87%. To examine the robustness of the above estimate, we used another method to estimate *ND* and *T*. We divided all genes in Fig 1A into four equal-interval bins according to their gene expression levels (i.e., all bins have the same log_10_RPM interval). Using the leftmost bin to estimate *T* and the rightmost bin to estimate *ND* yielded 1 –*ND*/*T* = 1–0.05/0.42 = 88%. Note that both of the above estimates of the fraction of deleterious ATI are conservative because *T* is an underestimate of all ATI before selection (because some deleterious ATI could have been removed in lowly expressed genes) and *ND* is an overestimate of all nondeleterious ATI (because some deleterious ATI may not have been removed in highly expressed genes). The above finding is broadly consistent with our estimate that only approximately 4.4% of genes have evidence for different optimal TSSs in different cell types and explains why *cis*-elements of minor TSSs are generally evolutionarily unconserved.

Some authors distinguished between two types of TSSs: sharp and broad TSSs [1, 26, 41]. Transcription initiates almost exclusively from one of a few neighboring nucleotides at a sharp TSS but from any of a large segment of nucleotides at a broad TSS. In the GRO-cap data of human K562 cell line [41] that we analyzed, 53.6% of sharp TSSs and 88.6% of broad TSSs are minor TSSs. Because we found that almost all minor TSSs are nonadaptive, the above numbers suggest that most sharp TSSs as well as the vast majority of broad TSSs are nonadaptive.

Even though ATI arises primarily from molecular error and is thus stochastic, the magnitude of this error does not have to be entirely random. Our results provide unequivocal evidence for multiple forms of regulation of the magnitude. For instance, because of the common *trans*-environment for transcriptional initiation in a tissue/cell line, the negative correlation between the expression level of a gene and its TSS diversity in a tissue/cell line indicates that the magnitude of transcriptional initiation error is regulated by *cis*-factors such as various *cis*-elements analyzed. Because the *cis*-factors for a gene are constant across cell lines, the observation that the same gene has lower TSS diversities in cell lines where its expression levels are higher indicates that the magnitude of transcriptional initiation error is also regulated by *trans*-factors. Because not all genes have lower TSS diversities in one cell line than in another cell line, the magnitude of transcriptional initiation error must also be regulated by interactions between *cis*- and *trans*-factors. The various trends we observed (**Figs 1–4**) strongly suggest that these regulations have been shaped by natural selection against transcriptional initiation error.

The disadvantage of using a particular minor TSS of a gene when compared with the use of the major TSS not only depends on the gene expression level but may also vary by the position of the minor TSS relative to the major TSS. One might think that using minor TSSs upstream of the major TSS is less deleterious than using minor TSSs downstream of the major TSS because using an upstream TSS makes the transcript longer, so it is unlikely to cause a disruption of any regulatory sequence that is supposed to be in the transcript from the major TSS. This prediction, however, may not be correct, because extending the 5′ UTR could cause the appearance of uORFs, which often interfere with the translation of the functional ORF [42, 43]. In the future, it will be interesting to study how fitness is altered by the use of minor TSSs at different positions. From the FANTOM TSS data, a substantial proportion of TSSs are found in RNA genes or intergenic regions, most of which are at the 5′ ends of genes encoding long noncoding RNAs [44] or microRNAs [45]. It will be important to verify whether our results on TSSs of protein-coding genes extend to RNA genes.

Our results on ATI echo recent findings about a number of phenomena that increase transcriptome diversity, including alternative polyadenylation [31], alternative splicing [46, 47], and several forms of RNA editing [48–50]. They have all been shown to be largely the results of molecular errors instead of adaptive regulatory mechanisms. Together, these findings reveal the astonishing imprecision of key molecular processes in the cell, contrasting the common view of an exquisitely perfected cellular life [51]. Interestingly, protein synthesis, the step of gene expression following transcription, also shows prevalent variations such as the non-AUG translational initiation [52], use of uORFs [53], and stop-codon readthrough [54]. It remains to be seen whether these variations that increase proteome diversity are also largely molecular errors.

That ATI is generally nonadaptive does not preclude the existence of a minority of cases of ATI that are beneficial [16, 20, 25]. However, identifying these few beneficial ATI cases will be challenging. Our past studies of RNA editing [49, 55] suggest that evolutionarily conserved TSSs may be beneficial, and future studies can prioritize those TSSs in the search for adaptive ATI.

## Materials and methods

### TSSs

The CAGE-seq data from human cell lines and tissues and mouse tissues were downloaded from FANTOM5 phase1.3 (http://fantom.gsc.riken.jp/5/datafiles/phase1.3/). We analyzed five human cell lines, as well as 11 tissues (10 specific tissues plus a mixed one) from each of the two species (**S1 Table**). FANTOM5 phase 1.3’s TSS annotation already clustered original TSSs into TSS peaks (a peak is a short genomic region that could include more than one original TSS) by the decomposition peak identification (DPI) algorithm upon the removal of technical noise [7]. For protein-coding genes, we found that only 0.0082% of adjacent peaks are <5 nucleotides apart, and only 0.14% of adjacent peaks are <10 nucleotides apart. The mean distance between adjacent peaks is 1,846 nucleotides, and the median is 135 nucleotides. The permissive and robust CAGE peaks identified by the original authors through rigorous filtering [7] were used in our analyses. Both types of peaks were obtained by removing technical noise followed by applying DPI [7]. As a result, both peaks are reliable, with the only difference being that the tag evidence thresholds are higher for robust peaks. In total, there were 1,048,124 permissive and 184,827 robust peaks identified from 975 human samples and 652,860 permissive and 116,227 robust peaks identified from 399 mouse samples, respectively. Because the robust set misses most TSSs (e.g., >80% in human) identified by high-throughput sequencing, we primarily used the permissive TSSs unless noted. TSS was defined as the position within a CAGE peak region with the highest total number of CAGE tags across all samples in FANTOM5 phase1.3.

Human (hg19 and hg38) and mouse (mm9) genomic annotations were downloaded from Ensembl (http://useast.ensembl.org/index.html). We focused our analysis on protein-coding genes, including 20,745 human genes and 22,745 mouse genes. A CAGE peak whose 5′ end is within 500 bp of the 5′ end of a gene is considered to belong to the gene if the peak is on the same strand as the gene [7]. Peaks mapped to more than one gene were removed. The total CAGE tags within a peak were considered as the tags of the representative TSS in that peak. The major TSS of a gene in a tissue or cell line is defined as the most frequently used TSS of the gene in the given tissue or cell line, while all other TSSs are considered minor.

In addition, we analyzed two independent 5′-end RNA-seq data: GRO-cap and TSS-seq. GRO-cap is the global nuclear run-on sequencing method that enriches for 5′-7meGTP-capped RNAs [41]. It differs from CAGE-seq in that it can also detect the TSSs of unstable RNAs. We analyzed the GRO-cap data from the human cell line K562 [41] to allow a direct comparison with the CAGE-seq-based result on the same cell line. To compare CAGE-seq and GRO-cap results fairly, we assigned GRO-cap reads to TSSs annotated in FANTOM5 phase 1.3 before computing TSS diversity. TSS-seq is another independent method for identifying TSSs and measuring their fractional usages [56]. TSSs and reads from TSS-seq [56] for 26 human cell lines and 16 human tissues (**S1 Table**) were downloaded from DBTSS (https://dbtss.hgc.jp/). The analyses of CAGE-seq and TSS-seq reads were the same except that the reference genome hg38 was used in TSS-seq read mapping by the original authors [56].

### Measures of TSS diversity and gene expression level

Simpson and Shannon indices of TSS diversity for a gene are respectively defined by $1–\sum_{\text{i=1}}^{\text{S}}p_{\text{i}}^{2}$and $–\sum_{\text{i=1}}^{\text{S}}p_{i}\text{ln}p_{\text{i}}$ where *S* is the number of TSSs in the gene and *p*_i_ is the proportion of RNA molecules of the gene that use the *i*th TSS. To measure the difference in TSS usage for a gene between CAGE-seq samples A and B, we used a net correlational distance defined by *d*_AB_− 0.5*d*_A_− 0.5*d*_B_. Here, *d*_AB_ equals 1 minus Pearson’s correlation coefficient between samples A and B in the fractional uses of all TSSs of the gene, *d*_A_ is the same as *d*_AB_ except that the two samples used are two CAGE-seq bootstrap samples derived from sample A, and *d*_B_ is the same as *d*_AB_ except that the two samples used are two CAGE-seq bootstrap samples derived from sample B [31].

Because CAGE-seq sequences only the 5′ end of an mRNA, the expression level of a gene is proportional to the number of CAGE-seq reads mapped to the gene [57]. The expression level of a gene is computed by the total number of CAGE-seq reads mapped to all TSSs of the gene multiplied by 10^6^ and then divided by the total number of reads mapped to all TSSs of all genes in the sample. This is referred to as RPM. Although CAGE-seq data are comparable with the canonical RNA-seq in measuring gene expression, we also measured gene expression levels by canonical RNA-seq [38] for the same human universal sample included in the CAGE-seq data.

### Down-sampling

To remove the potential influence of unequal sequencing depths of different genes in a sample on our analyses, we conducted down-sampling analyses. Briefly, we randomly picked the same number of CAGE-seq reads from all genes. Unless otherwise noted, we randomly picked 10 CAGE-seq reads per gene for all genes with at least 10 reads. Although using original and down-sampled data usually yielded qualitatively similar results, results from down-sampled data are more reliable because of equal surveys of ATI among genes.

### Computer simulation

To rule out the possibility that the trends observed in Figs 1A, 1C and 2A are statistical artifacts, we performed computer simulations of the same number of random genes as the actual number of genes analyzed. We randomly generated each gene whose expression level (reflected by the total number of reads mapped to all TSSs of the gene) and relative TSS usages (reflected by the numbers of reads mapped to various TSSs of the gene) respectively follow the gene-expression–level distribution and relative TSS usage distribution of the real genes. Specifically, the total read number of a simulated gene was sampled from the collection of actual read numbers of all genes with replacement, while the numbers of reads mapped to the TSSs of the gene were multinomial random variables drawn according to the TSS usages of a randomly picked real gene. We then analyzed the read data from the simulated genes as if they were the actual data.

### Paralogs and orthologs

Human paralogous genes as well as human–mouse one-to-one orthologous genes were downloaded from Ensembl (release 89; May 2017). We obtained 3,678 human gene families, including 51,657 pairs of human paralogous protein-coding genes. When comparing between paralogs, we randomly selected from each gene family only one paralogous pair that has at least a 2-fold expression difference to allow a sufficient statistical power. Because of the expression level variation among tissues or cell lines, the included paralogous pairs in our analysis vary by tissue or cell line. The number of orthologous genes between human and mouse is 16,805. The one-to-one orthologous CAGE peaks (i.e., orthologous TSSs) between human and mouse were obtained from FANTOM5 phase 1.3 (http://fantom.gsc.riken.jp/5/datafiles/phase1.3/extra/CAGE_peaks_Cross_species_projection/). When comparing TSSs between human and mouse orthologs, we set zero usage in mouse for TSSs that are found only in human and vice versa.

### *Cis*-elements of core promoters and the conservation score

The structure of a human core promoter with positions and consensus sequences of *cis*-elements [58] analyzed in this study is shown in **Fig 5A**. Following a previous study [58], we used the consensus sequences to search three *cis*-elements around TSS (+1); BRE was searched in [**–**50, –1], TATA box was searched in [**–**40, –1], and INR was searched in [**–**6, +7]. Only 12% of TSSs were found to have at least one of the three motifs because most minor TSSs do not have any detected motif. The fraction of TSSs with at least one motif tends to decrease with the TSS usage rank. All detected copies of a motif associated with a TSS were considered. To examine the evolutionary conservation of the *cis*-elements, we downloaded from UCSC (http://hgdownload.cse.ucsc.edu/goldenpath/hg19/phastCons46way/placentalMammals/) PhastCons scores computed from genome alignments of 46 placental mammals, including the human (hg19).

## Supporting information

S1 Fig Results in Fig 1 are robust to variations of the analyses. (A) Spearman's correlations between gene expression level and Simpson index of TSS diversity when only those TSSs that are within 500 bp from annotated TSSs of each gene are considered. (B) Spearman's correlations between gene expression level and Simpson index of TSS diversity when only robust TSSs of each gene are considered. (C–E) Spearman's correlations between gene expression level and Simpson index of TSS diversity when all TSSs located within 1 kb (C), 5 kb (D), or 10 kb (E) upstream of the most upstream TSS annotated are considered. (F) Spearman's correlation between gene expression level and Simpson index of TSS diversity in the human universal sample when gene expression levels are measured by RNA-seq. In (A)–(E), gray squares and black triangles show the correlations on the basis of the original and down-sampled data, respectively. Sample IDs listed on the *x*-axis refer to those in S1 Table. In (A), *P* < 10^−28^ in all cases. In (B), for the original data, *P* < 0.05 except for tissues #6 and #10; for down-sampled data, *P* < 0.05 except for tissue #2. In (C)–(E), *P* < 10^−39^ in all cases. In (F), the gray and black *ρ* and *P* are based on the original and down-sampled data, respectively. ID, identifier; RNA-seq, RNA sequencing; TSS, transcription start site.
(PDF)

S2 Fig **Spearman's correlations between the Simpson (A) or Shannon (B) index of TSS diversity and gene expression level in various down-sampled human CAGE-seq data.** We randomly sampled 5, 10, 20, 40, or 80 reads per gene from genes with at least that many reads. Gray squares and black triangles show the correlations on the basis of the original and down-sampled data, respectively. All correlations in down-sampled data are significantly negative (*P* < 0.05). Sample IDs listed on the *x*-axis refer to those in S1 Table. CAGE, cap analysis gene expression; CAGE-seq, CAGE sequencing; ID, identifier; TSS, transcription start site.
(PDF)

S3 Fig **Spearman's correlation (ρ) between the Simpson index of TSS diversity and expression level in human TSS-seq (A) and GRO-cap (B) samples.** In (A), gray squares and black triangles show the correlations on the basis of the original and down-sampled data, respectively. ρ is significantly negative (*P* < 10^−6^) for 40 of the 42 samples investigated. Sample IDs listed on the *x*-axis refer to those in S1 Table. In (B), each dot represents a gene. ρ and associated *P*-value are presented for the original data (gray) and down-sampled data (black), respectively. The sample used in (B) is K562 cell line. GRO-cap, 5′ global run-on sequencing; ID, identifier; TSS, transcription start site.
(PDF)

S4 Fig Within a pair of human paralogous genes, the relatively highly expressed gene tends to have lower TSS diversity than the relatively lowly expressed gene. (A) Simpson index of TSS diversity in the human universal sample for each member of a paralogous gene pair. Each dot represents a paralogous pair. Dots above and below the diagonal are colored red and blue, respectively. Numbers of red and blue dots are respectively indicated in the corresponding color. *P*-value is from a binomial test of the null hypothesis of equal numbers of red and blue dots. (B) Fraction of paralogous gene pairs for which the Simpson (upper panel) or Shannon (lower panel) index of the relatively lowly expressed gene exceeds that of the relatively highly expressed gene. Gray squares and black triangles show the results from the original and down-sampled data, respectively. Sample IDs listed on the *x*-axis of (B) refer to those in S1 Table. All fractions from down-sampled data are significantly greater than 0.5 (*P* < 0.02). ID, identifier; TSS, transcription start site.
(PDF)

S5 Fig **Spearman's correlation (ρ) between the Simpson (A) or Shannon (B) index of TSS diversity and expression level in mouse CAGE-seq data.** The diversity indices are calculated from down-sampled data. All correlations are significantly negative (*P* < 0.05) except for samples #2 and #4 in the category of 80 reads of both panels. Sample IDs listed on the *x*-axis refer to those in S1 Table. CAGE, cap analysis gene expression; CAGE-seq, CAGE sequencing; ID, identifier; TSS, transcription start site.
(PDF)

S6 Fig Results in Fig 2 are robust to variations of the analyses. Spearman's rank correlation between the expression level of a human gene and the fractional uses of its TSSs when only TSSs within 500 bp from any annotated TSS of a gene are considered (A), when only robust TSSs are considered (B), when TSSs located within 1 kb (C), 5 kb (D), or 10 kb (E) upstream of the most upstream TSS annotated are considered, or when gene expression level is measured by RNA-seq (F). The correlations for TSSs with a particular rank are calculated using the genes that have at least that particular number of TSSs. All *P* < 0.01. Sample IDs listed on the *x*-axis refer to those in S1 Table. Results in (A)–(E) are based on down-sampled data. In (F), each dot depicts the original data from one gene. Shown in gray and black are respectively the *ρ* (and *P*) computed from the original and down-sampled data. ID, identifier; RNA-seq, RNA sequencing; TSS, transcription start site.
(PDF)

S7 Fig Spearman's rank correlations between the expression level of a gene and the fractional uses of its TSSs in human TSS-seq samples. The correlation for TSSs with a particular rank is calculated using the genes that have at least that particular number of TSSs. All correlations for rank #1 are significantly positive (*P* < 0.05) except for tissue #16. All correlations for ranks #2, #3, and #4 are significantly negative (*P* < 10^−54^). Sample IDs listed on the *x*-axis refer to those in S1 Table. Down-sampled data are used here. ID, identifier; TSS, transcription start site; TSS-seq, TSS sequencing.
(PDF)

S8 Fig Spearman's rank correlations between the expression level of a gene and the fractional uses of its TSSs in human or mouse CAGE-seq data. (A) Spearman's rank correlations between the expression level of a gene and the fractional uses of its TSSs among human genes with at least four TSSs in each sample. (B) Spearman's rank correlations between the expression level of a gene and the fractional uses of its TSSs in mouse tissues. The correlation for TSSs with a particular rank is calculated using the genes that have at least that particular number of TSSs. (C) Spearman's rank correlations between the expression level of a gene and the fractional uses of its TSSs among mouse genes with at least four TSSs in each sample. In all panels, the correlations for rank #1 are significantly positive (*P* < 10^−3^) in down-sampled data. All correlations for ranks #2, #3, and #4 are significantly negative (*P* <10^−3^). Sample IDs listed on the *x*-axis refer to those in S1 Table. CAGE, cap analysis gene expression; CAGE-seq, CAGE sequencing; ID, identifier; TSS, transcription start site.
(PDF)

S9 Fig Fractional uses of TSSs in human paralogous genes. (A) Fractional uses of TSSs of ranks #1 to #4 in the relatively lowly expressed and relatively highly expressed members of each paralogous gene pair in the human universal sample. Each dot represents a paralogous gene pair. Dots above and below the diagonal are colored red and blue, respectively. Numbers of red and blue dots are respectively indicated. *P*-value is based on a binomial test of the null hypothesis of equal numbers of red and blue dots. (B) Proportion of paralogous gene pairs for which the fractional usage of a ranked TSS is higher in the more highly expressed paralog. *P* < 0.05 in all down-sampled cases. Sample IDs listed on the *x*-axis refer to those in S1 Table. ID, identifier; TSS, transcription start site.
(PDF)

S10 Fig Proportion of genes with a positive Spearman's correlation among human cell lines between the fractional uses of TSSs of the global ranks #1 to #4 in a cell line and the gene expression level in the cell line. The global rank of a TSS is determined using all reads from the five human cell lines. All *P*-values (binomial tests) are less than 0.05. Results are from down-sampled data. TSS, transcription start site.
(PDF)

S11 Fig Evolutionary conservations of *cis*-elements of human core promoters. Mean PhastCons scores of *cis*-elements of global major TSSs, *cis*-elements of global upstream minor TSSs, and upstream pseudoelements for INR (A), BRE (B), and TATA box (C). Upstream minor *cis*-elements refer to the elements corresponding to minor TSSs that are upstream of the major TSS. Upstream pseudoelements are identified from the complementary sequence of [–50, +7]. In each panel, the mean PhastCons score is significantly different (*P* < 0.05, Mann–Whitney U test) between any pair of the three bins. Error bars show the standard error. BRE, TFIIB recognition element; INR, initiator; TSS, transcription start site.
(PDF)

S1 Table 5′-end sequencing data sets used in this study. (PDF)

S2 Table Correlations between gene expression level and TSS diversity or relative usage of a ranked TSS in simulated data. TSS, transcription start site.
(PDF)

## Abbreviations

ATI: alternative transcriptional initiation
BRE: TFIIB recognition element
CAGE-seq: cap analysis gene expression sequencing
CDS: coding sequence
deepCAGE: deep cap analysis gene expression
DPE: downstream promoter element
DPI: decomposition peak identification
FANTOM: functional annotation of the mouse/mammalian genome
GRO-cap: 5*′* global run-on sequencing
ID: identifier
INR: initiator
IRES: internal ribosome entry site
LEF1: lymphoid enhancer binding factor 1
RNA-seq: RNA sequencing
RPM: reads mapped to the gene per million reads
RUNX1: runt-related transcription factor 1
TSS: transcription start site
TSS-seq: TSS sequencing
uORF: upstream open read frame
UTR: untranslated region
Wnt: Wingless/Integrated
