# Supplementary figures and images for: Evidence that alternative transcriptional initiation is largely nonadaptive
Source: PLoS Biol. 2019 Mar 18;17(3):e3000197. doi: 10.1371/journal.pbio.3000197 (PMC6438578; doi:10.1371/journal.pbio.3000197)

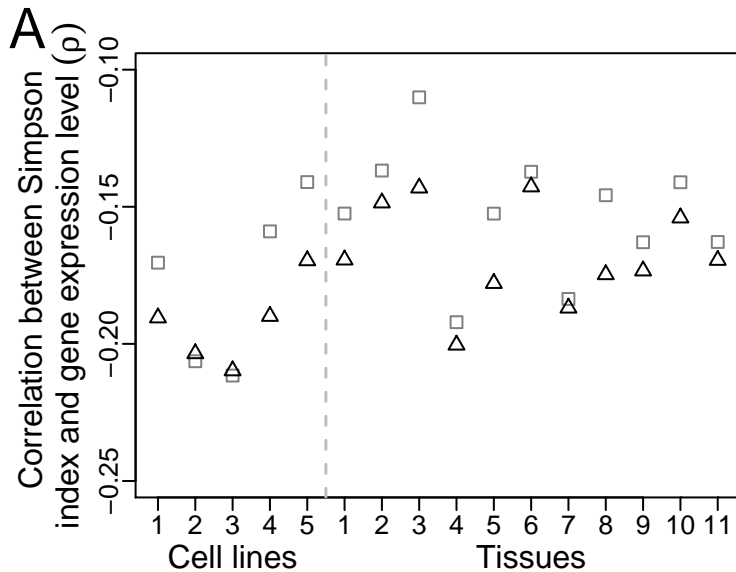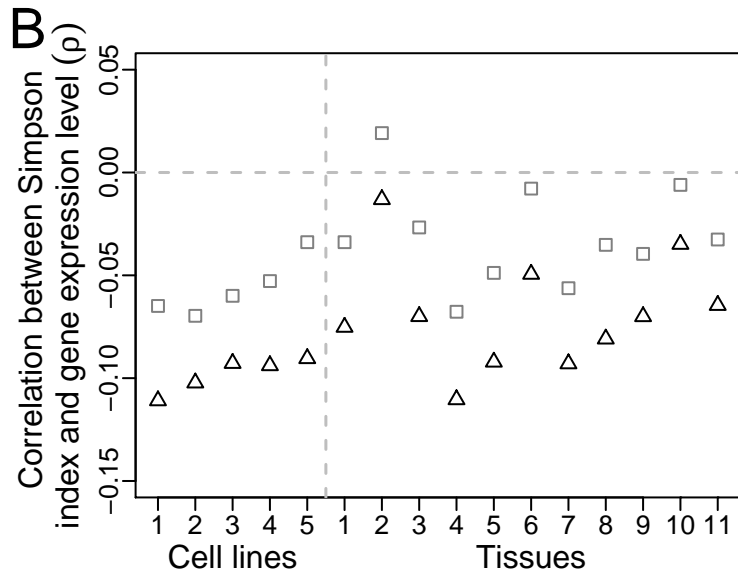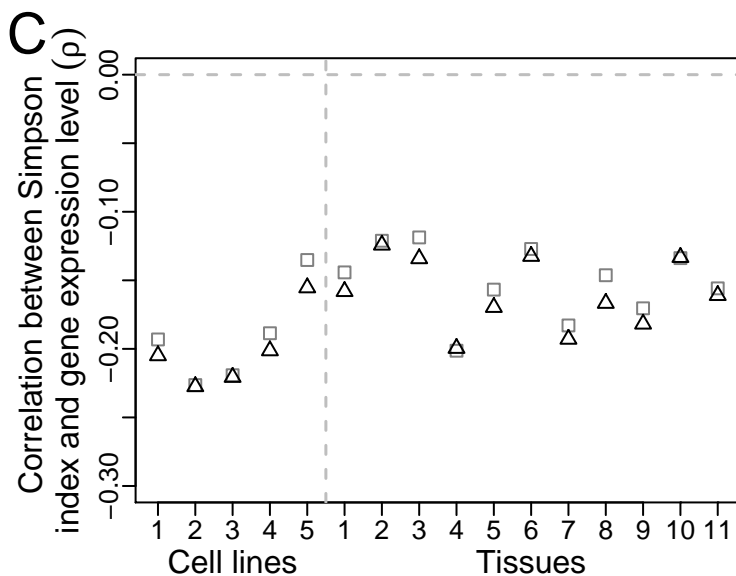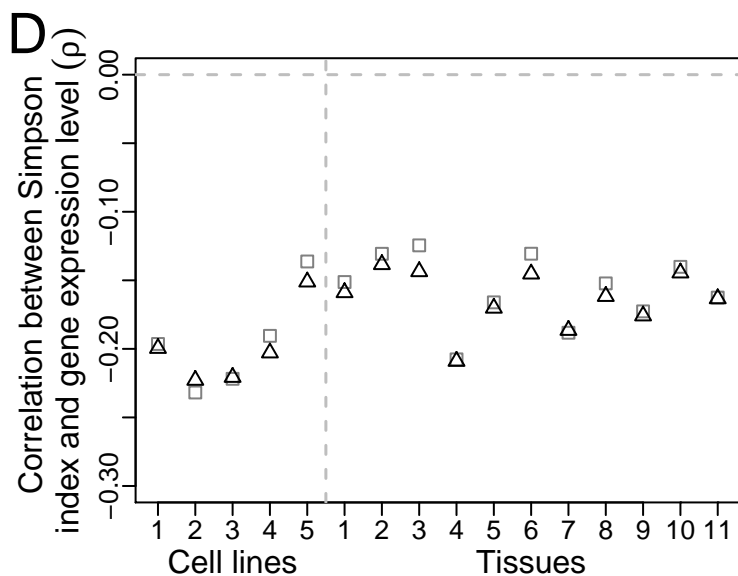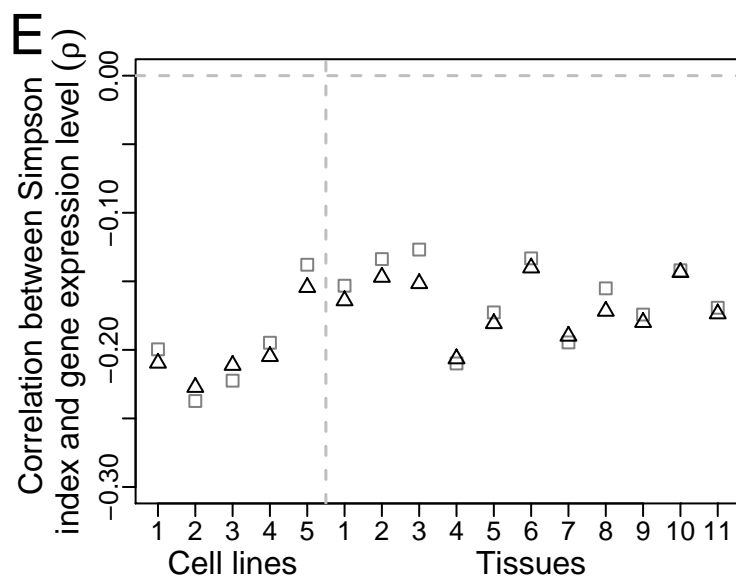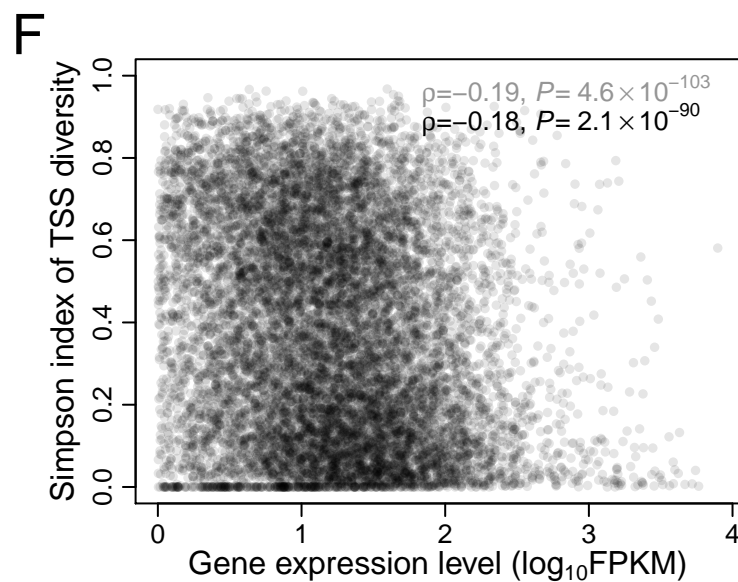

Supplement: S1 Fig — (A) Spearman's correlations between gene expression level and Simpson index of TSS diversity when only those TSSs that are within 500 bp from annotated TSSs of each gene are considered. (B) Spearman's correlations between gene expression level and Simpson index of TSS diversity when only robust TSSs of each gene are considered. (C–E) Spearman's correlations between gene expression level and Simpson index of TSS diversity when all TSSs located within 1 kb (C), 5 kb (D), or 10 kb (E) upstream of the most upstream TSS annotated are considered. (F) Spearman's correlation between gene expression level and Simpson index of TSS diversity in the human universal sample when gene expression levels are measured by RNA-seq. In (A)–(E), gray squares and black triangles show the correlations on the basis of the original and down-sampled data, respectively. Sample IDs listed on the x-axis refer to those in S1 Table. In (A), P < 10−28 in all cases. In (B), for the original data, P < 0.05 except for tissues #6 and #10; for down-sampled data, P < 0.05 except for tissue #2. In (C)–(E), P < 10−39 in all cases. In (F), the gray and black ρ and P are based on the original and down-sampled data, respectively. ID, identifier; RNA-seq, RNA sequencing; TSS, transcription start site. (PDF) [file pbio.3000197.s001.pdf]

**A**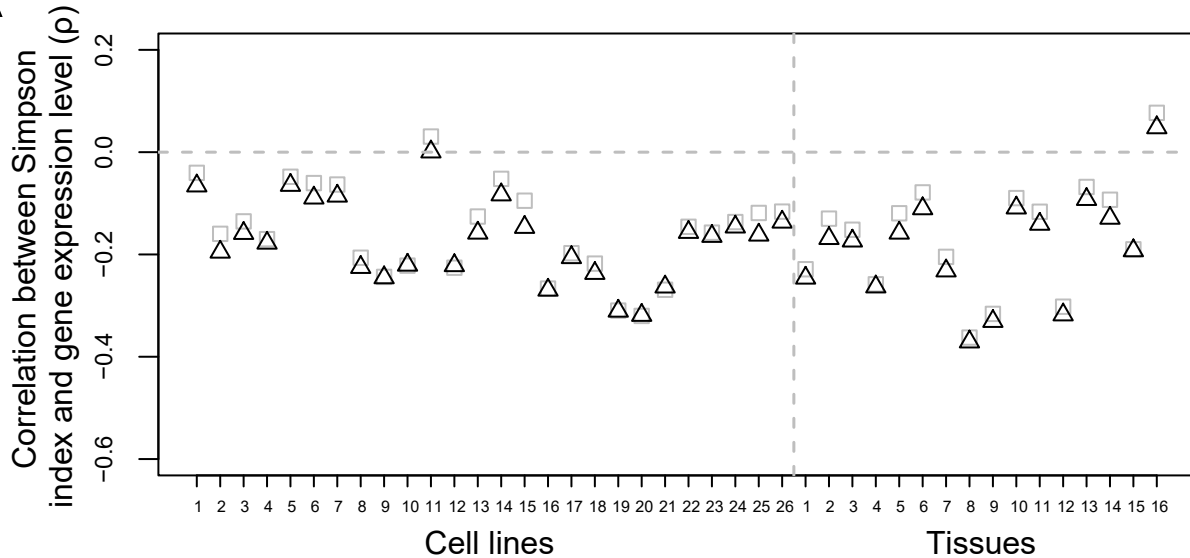**B**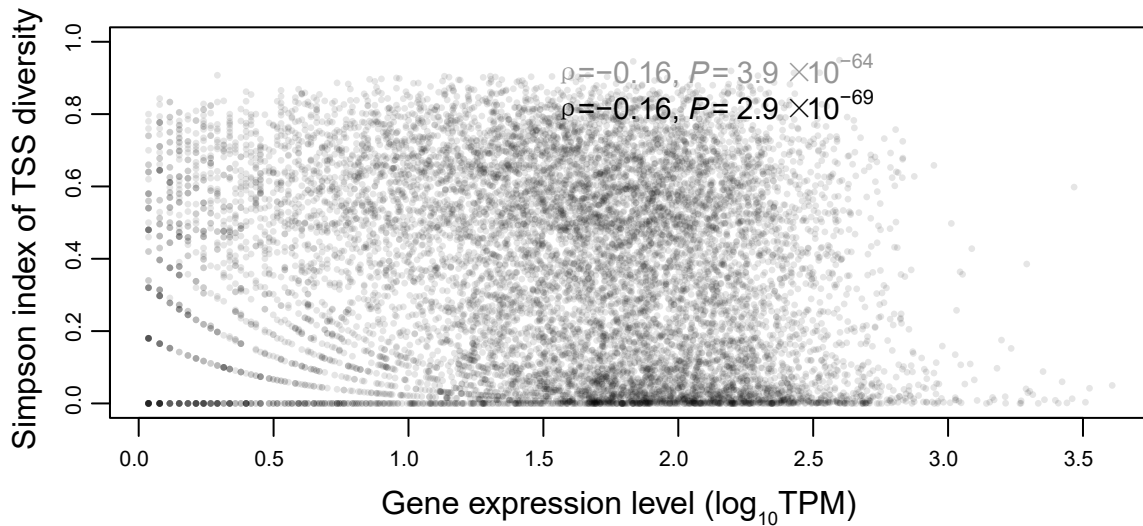

Supplement: S3 Fig — Spearman's correlation (ρ) between the Simpson index of TSS diversity and expression level in human TSS-seq (A) and GRO-cap (B) samples. In (A), gray squares and black triangles show the correlations on the basis of the original and down-sampled data, respectively. ρ is significantly negative (P < 10−6) for 40 of the 42 samples investigated. Sample IDs listed on the x-axis refer to those in S1 Table. In (B), each dot represents a gene. ρ and associated P-value are presented for the original data (gray) and down-sampled data (black), respectively. The sample used in (B) is K562 cell line. GRO-cap, 5′ global run-on sequencing; ID, identifier; TSS, transcription start site. (PDF) [file pbio.3000197.s003.pdf]

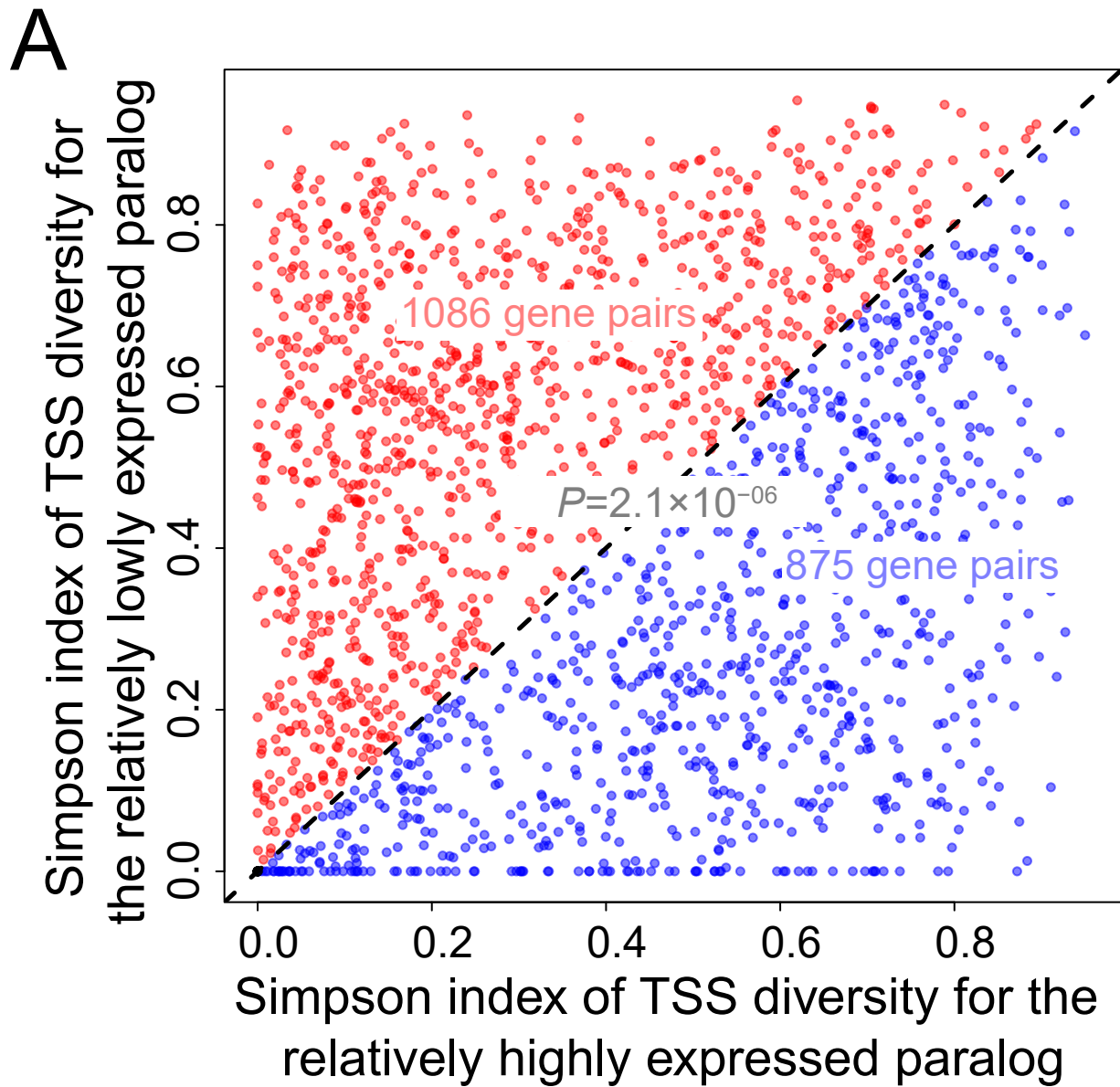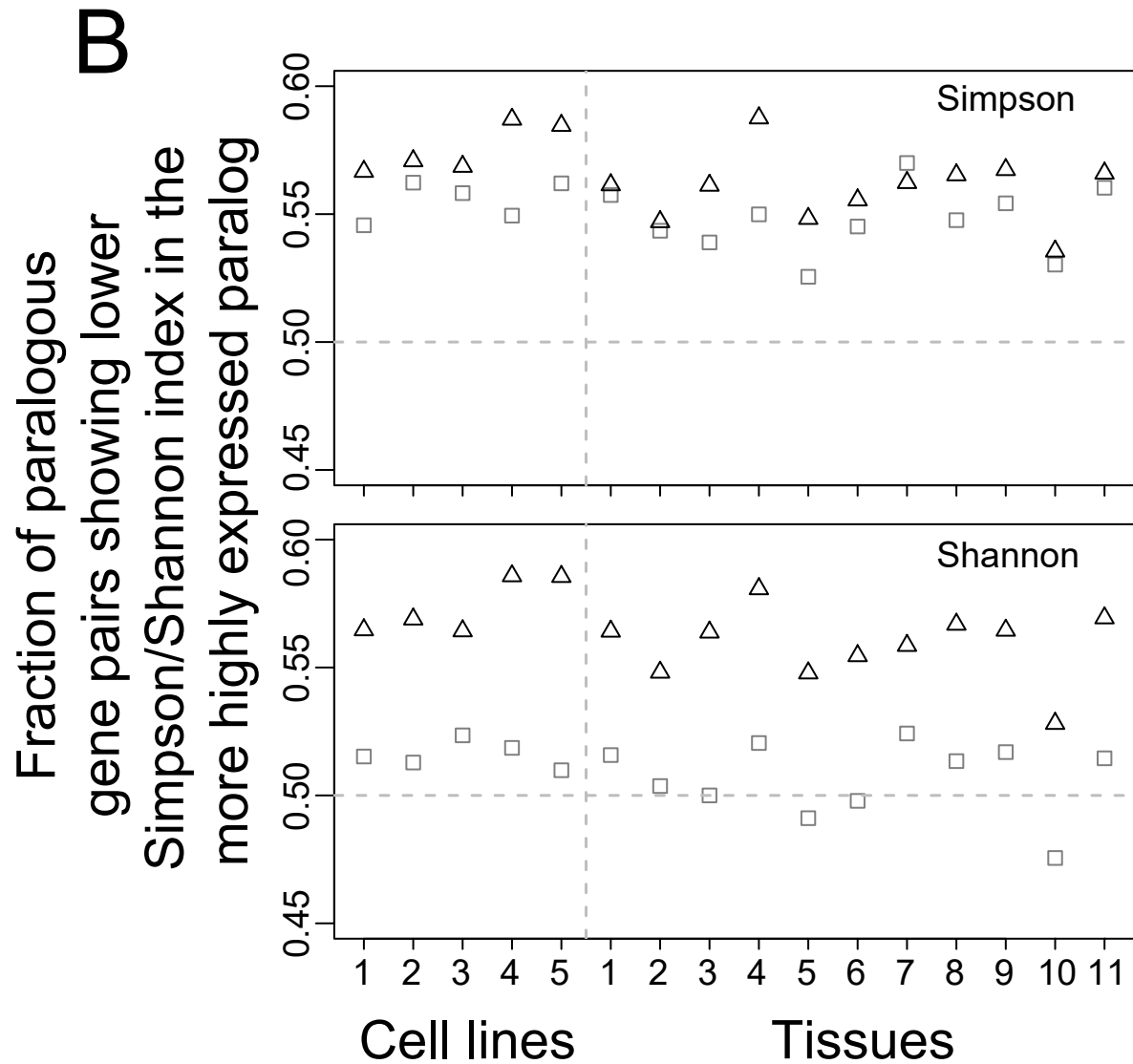

Supplement: S4 Fig — (A) Simpson index of TSS diversity in the human universal sample for each member of a paralogous gene pair. Each dot represents a paralogous pair. Dots above and below the diagonal are colored red and blue, respectively. Numbers of red and blue dots are respectively indicated in the corresponding color. P-value is from a binomial test of the null hypothesis of equal numbers of red and blue dots. (B) Fraction of paralogous gene pairs for which the Simpson (upper panel) or Shannon (lower panel) index of the relatively lowly expressed gene exceeds that of the relatively highly expressed gene. Gray squares and black triangles show the results from the original and down-sampled data, respectively. Sample IDs listed on the x-axis of (B) refer to those in S1 Table. All fractions from down-sampled data are significantly greater than 0.5 (P < 0.02). ID, identifier; TSS, transcription start site. (PDF) [file pbio.3000197.s004.pdf]

**A**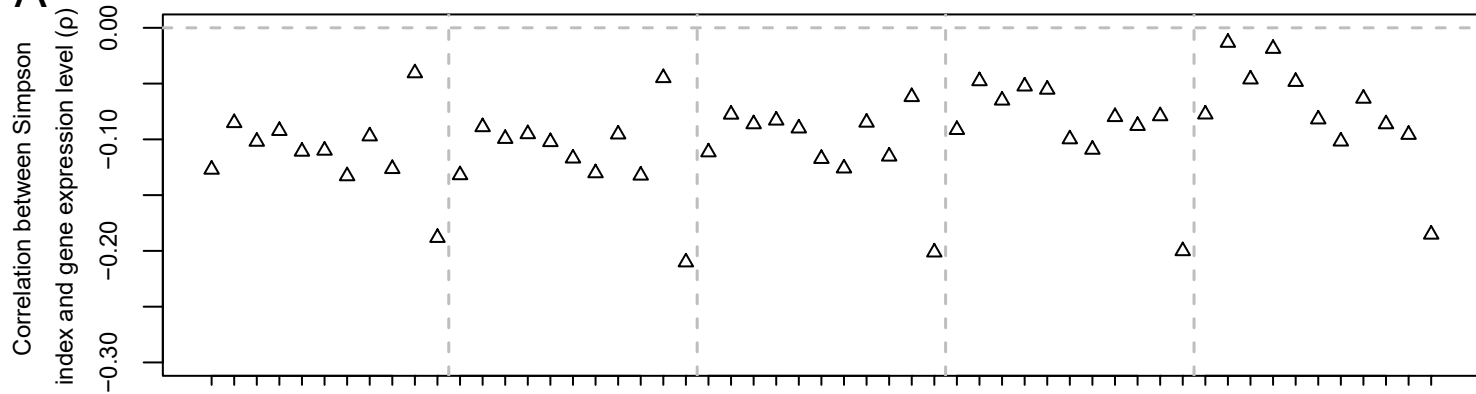**B**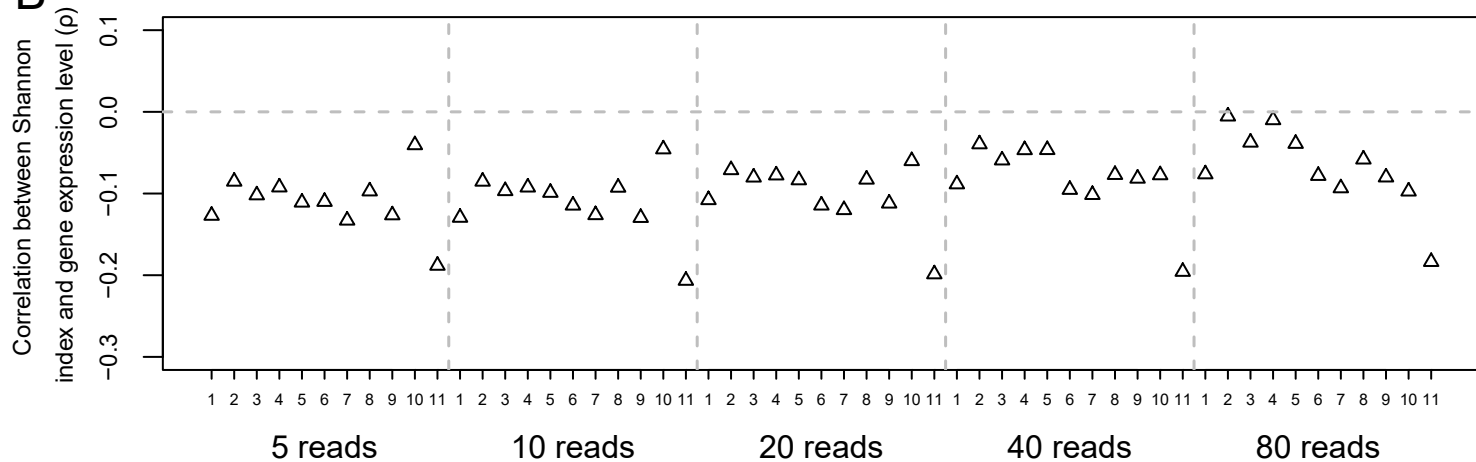

Supplement: S5 Fig — Spearman's correlation (ρ) between the Simpson (A) or Shannon (B) index of TSS diversity and expression level in mouse CAGE-seq data. The diversity indices are calculated from down-sampled data. All correlations are significantly negative (P < 0.05) except for samples #2 and #4 in the category of 80 reads of both panels. Sample IDs listed on the x-axis refer to those in S1 Table. CAGE, cap analysis gene expression; CAGE-seq, CAGE sequencing; ID, identifier; TSS, transcription start site. (PDF) [file pbio.3000197.s005.pdf]

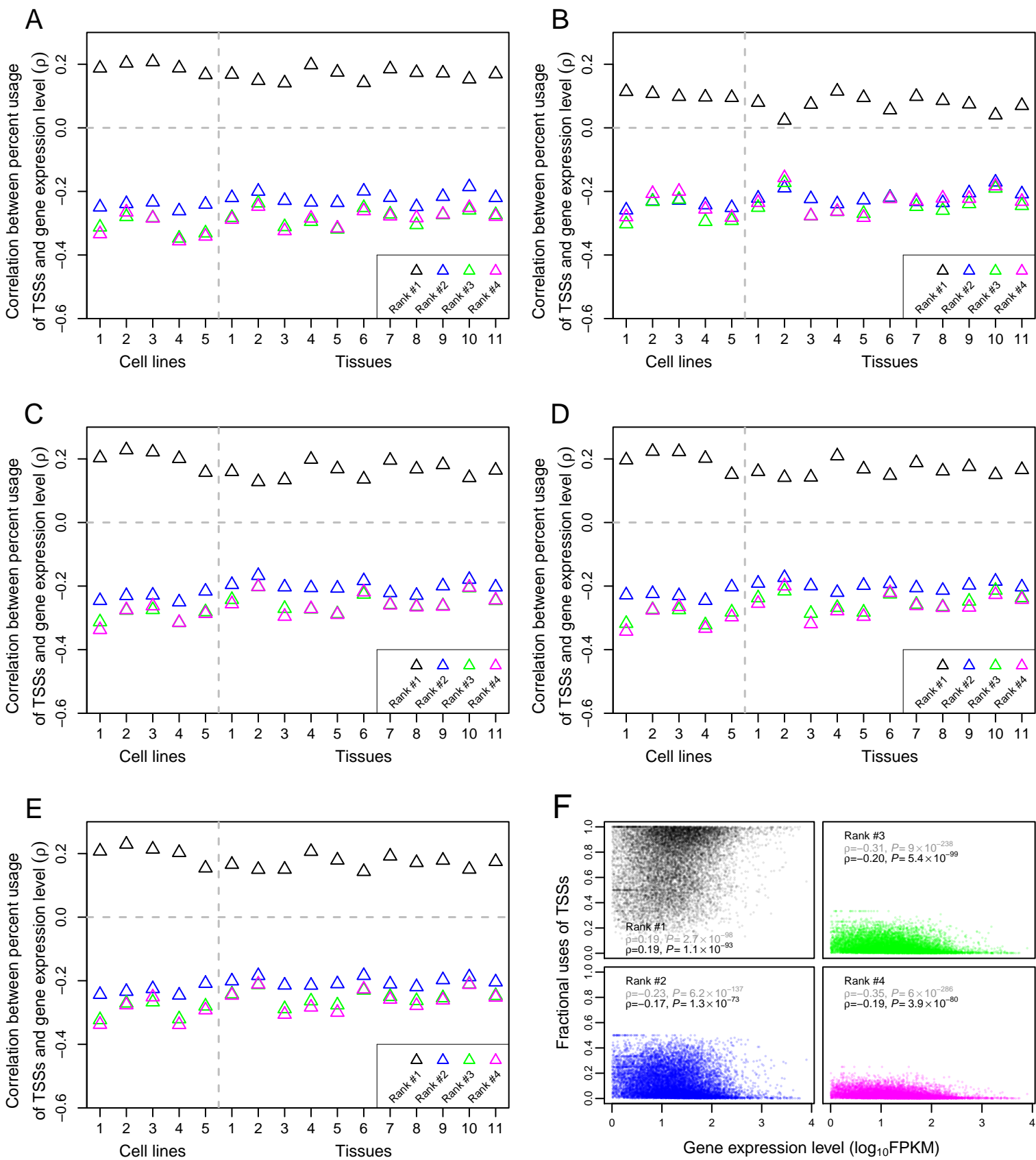

Supplement: S6 Fig — Spearman's rank correlation between the expression level of a human gene and the fractional uses of its TSSs when only TSSs within 500 bp from any annotated TSS of a gene are considered (A), when only robust TSSs are considered (B), when TSSs located within 1 kb (C), 5 kb (D), or 10 kb (E) upstream of the most upstream TSS annotated are considered, or when gene expression level is measured by RNA-seq (F). The correlations for TSSs with a particular rank are calculated using the genes that have at least that particular number of TSSs. All P < 0.01. Sample IDs listed on the x-axis refer to those in S1 Table. Results in (A)–(E) are based on down-sampled data. In (F), each dot depicts the original data from one gene. Shown in gray and black are respectively the ρ (and P) computed from the original and down-sampled data. ID, identifier; RNA-seq, RNA sequencing; TSS, transcription start site. (PDF) [file pbio.3000197.s006.pdf]

Correlation between percent usage  
of TSSs and gene expression level ( $\rho$ )

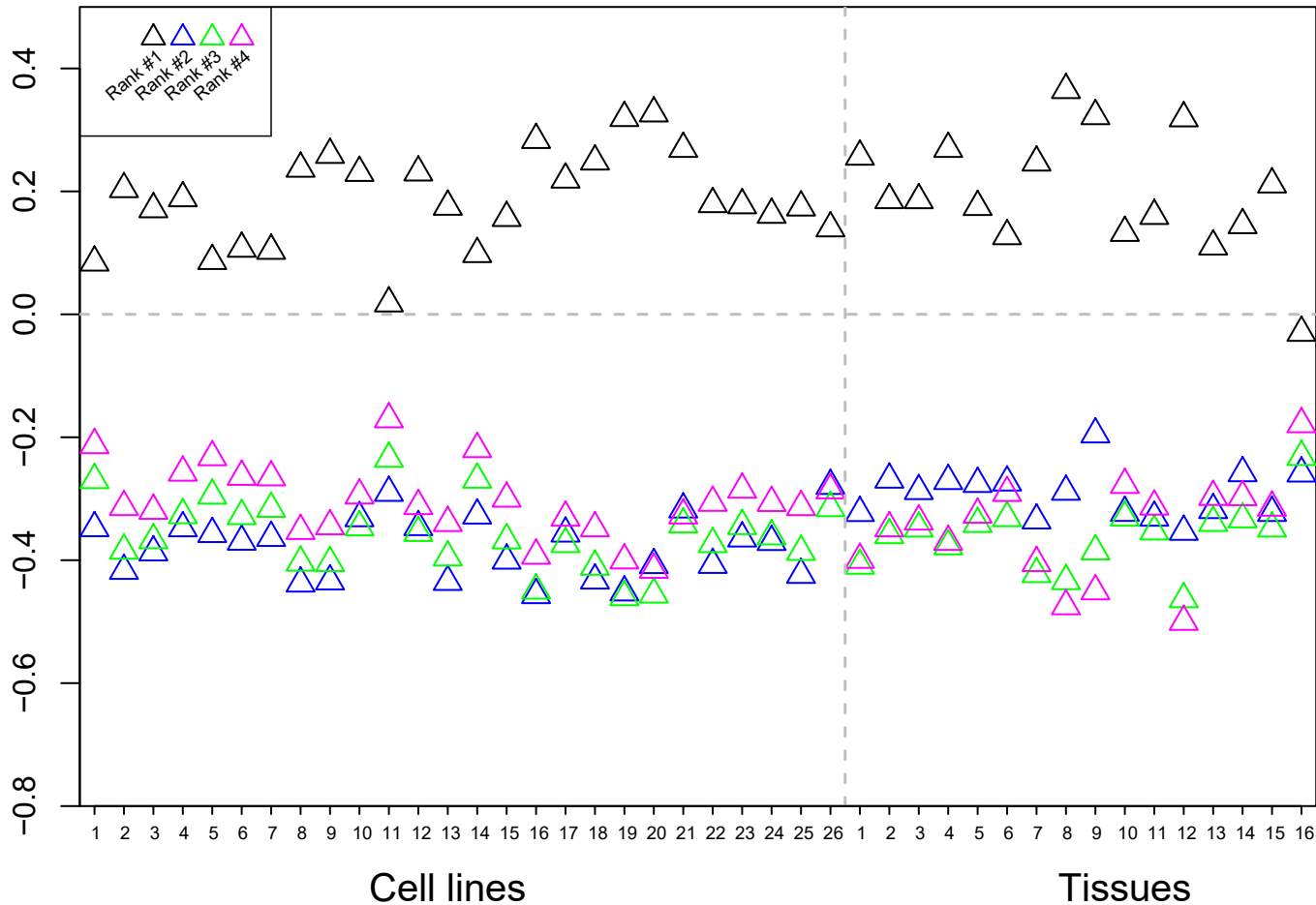

Supplement: S7 Fig — The correlation for TSSs with a particular rank is calculated using the genes that have at least that particular number of TSSs. All correlations for rank #1 are significantly positive (P < 0.05) except for tissue #16. All correlations for ranks #2, #3, and #4 are significantly negative (P < 10−54). Sample IDs listed on the x-axis refer to those in S1 Table. Down-sampled data are used here. ID, identifier; TSS, transcription start site; TSS-seq, TSS sequencing. (PDF) [file pbio.3000197.s007.pdf]

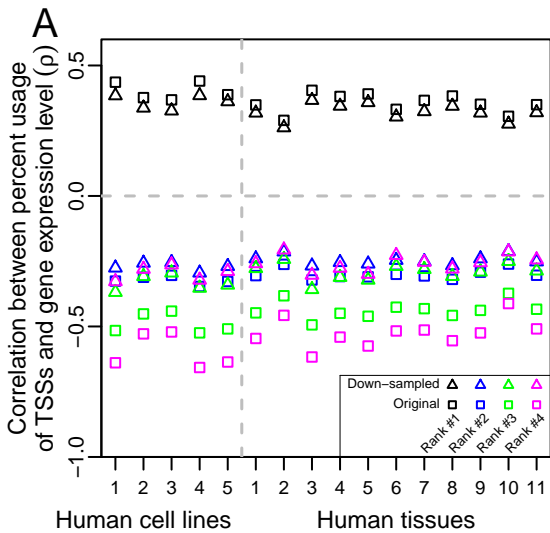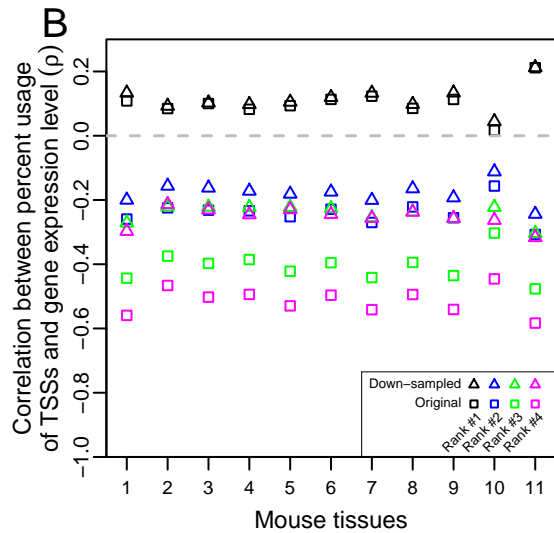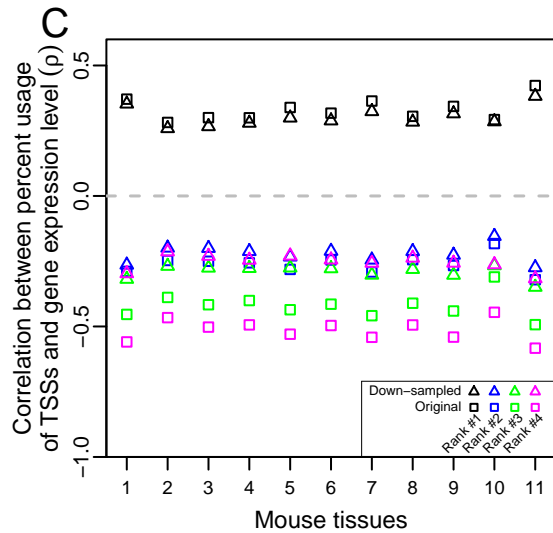

Supplement: S8 Fig — (A) Spearman's rank correlations between the expression level of a gene and the fractional uses of its TSSs among human genes with at least four TSSs in each sample. (B) Spearman's rank correlations between the expression level of a gene and the fractional uses of its TSSs in mouse tissues. The correlation for TSSs with a particular rank is calculated using the genes that have at least that particular number of TSSs. (C) Spearman's rank correlations between the expression level of a gene and the fractional uses of its TSSs among mouse genes with at least four TSSs in each sample. In all panels, the correlations for rank #1 are significantly positive (P < 10−3) in down-sampled data. All correlations for ranks #2, #3, and #4 are significantly negative (P <10−3). Sample IDs listed on the x-axis refer to those in S1 Table. CAGE, cap analysis gene expression; CAGE-seq, CAGE sequencing; ID, identifier; TSS, transcription start site. (PDF) [file pbio.3000197.s008.pdf]

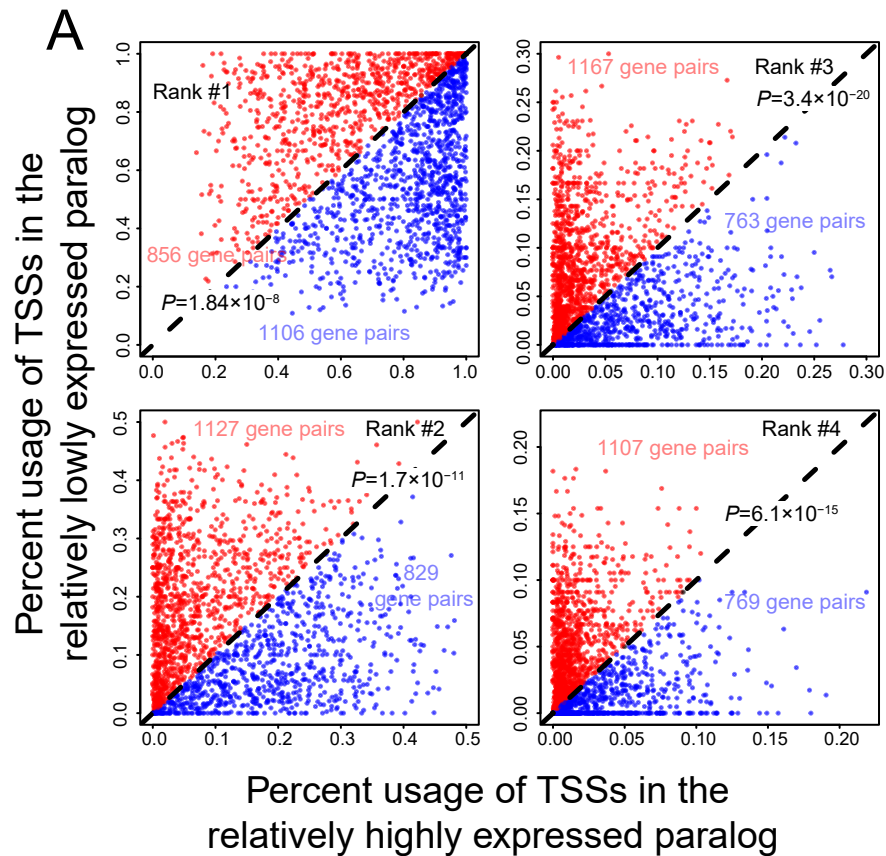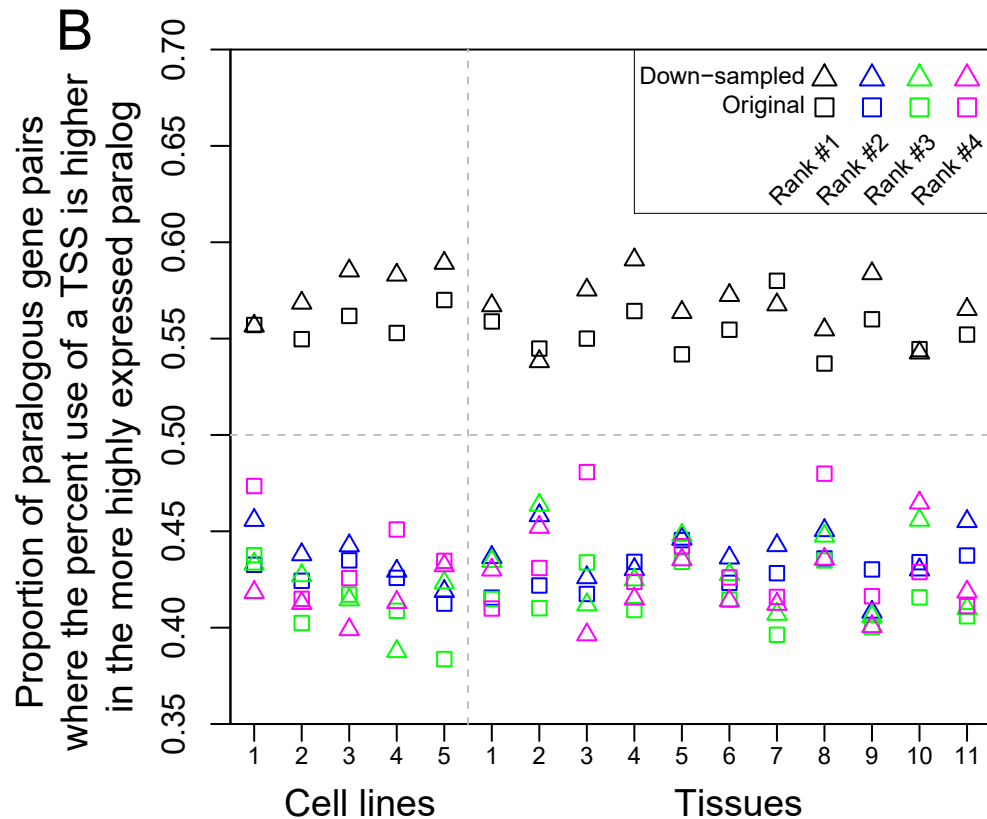

Supplement: S9 Fig — (A) Fractional uses of TSSs of ranks #1 to #4 in the relatively lowly expressed and relatively highly expressed members of each paralogous gene pair in the human universal sample. Each dot represents a paralogous gene pair. Dots above and below the diagonal are colored red and blue, respectively. Numbers of red and blue dots are respectively indicated. P-value is based on a binomial test of the null hypothesis of equal numbers of red and blue dots. (B) Proportion of paralogous gene pairs for which the fractional usage of a ranked TSS is higher in the more highly expressed paralog. P < 0.05 in all down-sampled cases. Sample IDs listed on the x-axis refer to those in S1 Table. ID, identifier; TSS, transcription start site. (PDF) [file pbio.3000197.s009.pdf]

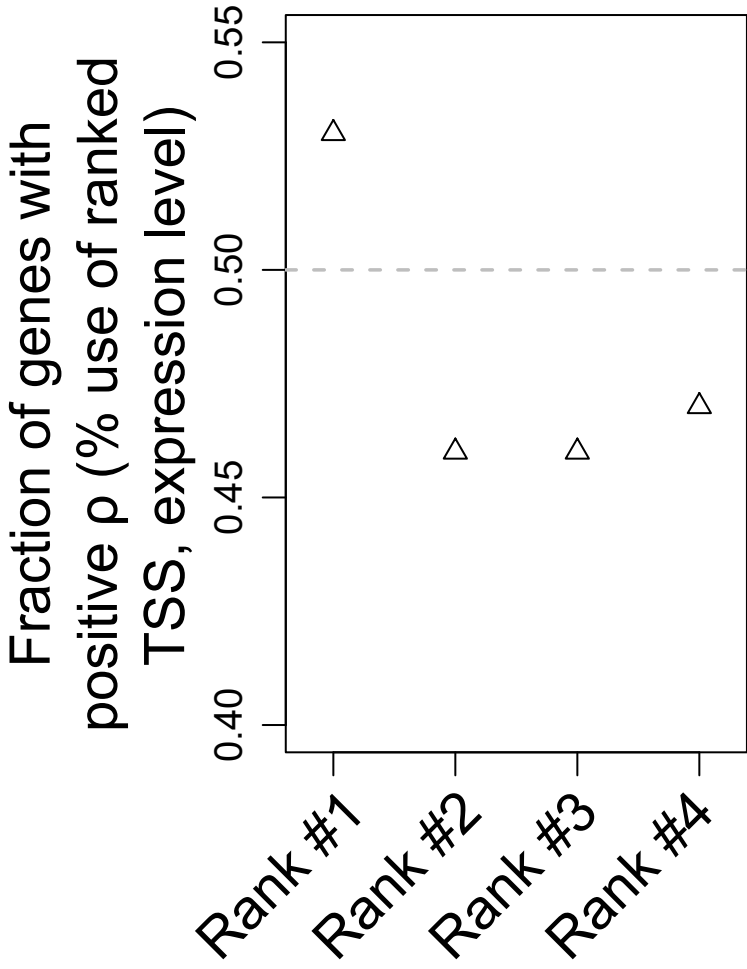

Supplement: S10 Fig — The global rank of a TSS is determined using all reads from the five human cell lines. All P-values (binomial tests) are less than 0.05. Results are from down-sampled data. TSS, transcription start site. (PDF) [file pbio.3000197.s010.pdf]

**A****INR**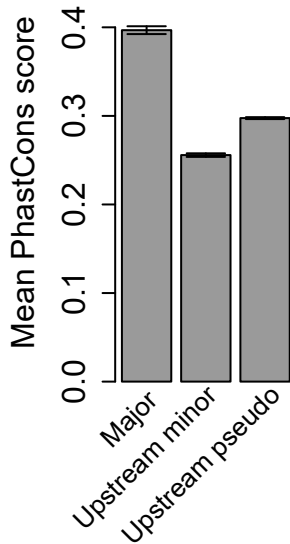**B****BRE**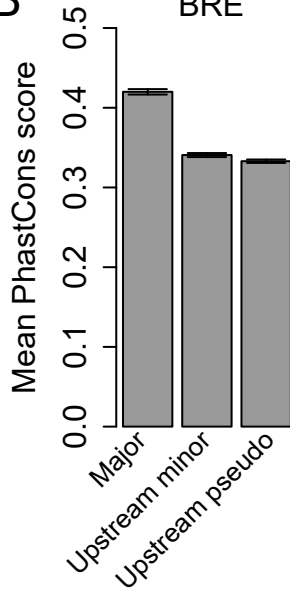**C****TATA**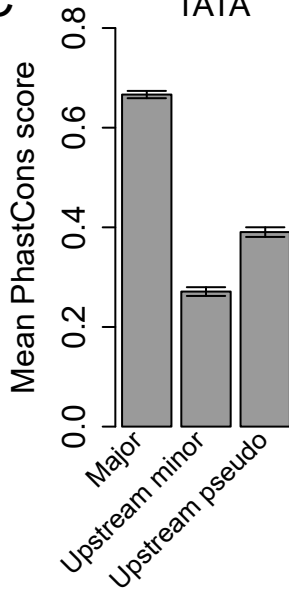

Supplement: S11 Fig — Mean PhastCons scores of cis-elements of global major TSSs, cis-elements of global upstream minor TSSs, and upstream pseudoelements for INR (A), BRE (B), and TATA box (C). Upstream minor cis-elements refer to the elements corresponding to minor TSSs that are upstream of the major TSS. Upstream pseudoelements are identified from the complementary sequence of [–50, +7]. In each panel, the mean PhastCons score is significantly different (P < 0.05, Mann–Whitney U test) between any pair of the three bins. Error bars show the standard error. BRE, TFIIB recognition element; INR, initiator; TSS, transcription start site. (PDF) [file pbio.3000197.s011.pdf]
