# Supplementary material for: Evidence that alternative transcriptional initiation is largely nonadaptive
Source: PLoS Biol. 2019 Mar 18;17(3):e3000197. doi: 10.1371/journal.pbio.3000197 (PMC6438578; doi:10.1371/journal.pbio.3000197)
Supplement: S2 Fig — Spearman's correlations between the Simpson (A) or Shannon (B) index of TSS diversity and gene expression level in various down-sampled human CAGE-seq data. We randomly sampled 5, 10, 20, 40, or 80 reads per gene from genes with at least that many reads. Gray squares and black triangles show the correlations on the basis of the original and down-sampled data, respectively. All correlations in down-sampled data are significantly negative (P < 0.05). Sample IDs listed on the x-axis refer to those in S1 Table. CAGE, cap analysis gene expression; CAGE-seq, CAGE sequencing; ID, identifier; TSS, transcription start site. (PDF) [file pbio.3000197.s002.pdf]

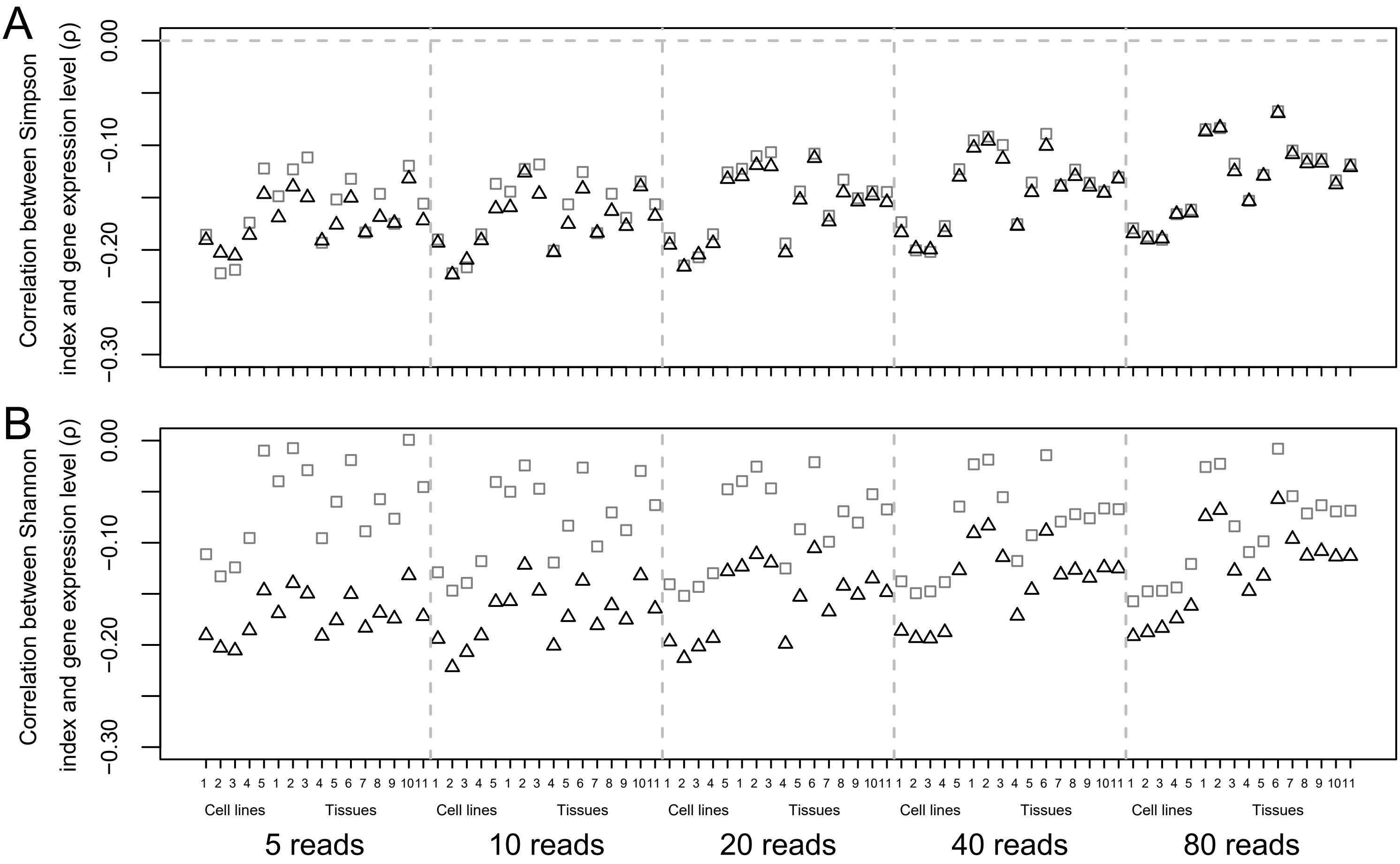
A
B
Correlation between Simpson index and gene expression level (ρ)
Correlation between Shannon index and gene expression level (ρ)
0.00
−0.10
−0.20
−0.30
1 2 3 4 5 1 2 3 4 5 6 7 8 9 10 11
Cell lines
Tissues
5 reads
10 reads
20 reads
40 reads
80 reads
