# Supplementary material for: Evidence that alternative transcriptional initiation is largely nonadaptive
Source: PLoS Biol. 2019 Mar 18;17(3):e3000197. doi: 10.1371/journal.pbio.3000197 (PMC6438578; doi:10.1371/journal.pbio.3000197)
Supplement: S1 Table — (PDF) [file pbio.3000197.s012.pdf]

## S1 Table. 5'-end sequencing datasets used in this study.

| Technique | Species | Database | Library | Sample ID | Sample | Type | Link |
|---|---|---|---|---|---|---|---|
| CAGE-seq | human | FANTOM5_phase1.3 | CNhs11275 | 1 | A549 | cell line | http://fantom.gsc.riken.jp/5/datafiles/phase1.3/basic/human.cell_line.hCAGE/ |
| CAGE-seq | human | FANTOM5_phase1.3 | CNhs12326 | 2 | HelaS3 | cell line | |
| CAGE-seq | human | FANTOM5_phase1.3 | CNhs12330 | 3 | HepG2 | cell line | |
| CAGE-seq | human | FANTOM5_phase1.3 | CNhs12335 | 4 | K562 | cell line | |
| CAGE-seq | human | FANTOM5_phase1.3 | CNhs11943 | 5 | MCF7 | cell line | |
| CAGE-seq | human | FANTOM5_phase1.3 | CNhs10616 | 1 | Bladder | tissue | http://fantom.gsc.riken.jp/5/datafiles/phase1.3/basic/human.tissue.hCAGE/ |
| CAGE-seq | human | FANTOM5_phase1.3 | CNhs10617 | 2 | Brain | tissue | |
| CAGE-seq | human | FANTOM5_phase1.3 | CNhs10621 | 3 | Heart | tissue | |
| CAGE-seq | human | FANTOM5_phase1.3 | CNhs10622 | 4 | Kidney | tissue | |
| CAGE-seq | human | FANTOM5_phase1.3 | CNhs10624 | 5 | Liver | tissue | |
| CAGE-seq | human | FANTOM5_phase1.3 | CNhs10625 | 6 | Lung | tissue | |
| CAGE-seq | human | FANTOM5_phase1.3 | CNhs10626 | 7 | Ovary | tissue | |
| CAGE-seq | human | FANTOM5_phase1.3 | CNhs10629 | 8 | Muscle | tissue | |
| CAGE-seq | human | FANTOM5_phase1.3 | CNhs10631 | 9 | Spleen | tissue | |
| CAGE-seq | human | FANTOM5_phase1.3 | CNhs10632 | 10 | Testis | tissue | |
| CAGE-seq | human | FANTOM5_phase1.3 | CNhs10612 | 11 | Universal | tissue | |
| CAGE-seq | mouse | FANTOM5_phase1.3 | CNhs10481 | 1 | Bladder | tissue | http://fantom.gsc.riken.jp/5/datafiles/phase1.3/basic/mouse.tissue.hCAGE/ |
| CAGE-seq | mouse | FANTOM5_phase1.3 | CNhs10473 | 2 | Cortex | tissue | |
| CAGE-seq | mouse | FANTOM5_phase1.3 | CNhs10482 | 3 | Diencephalon | tissue | |
| CAGE-seq | mouse | FANTOM5_phase1.3 | CNhs10478 | 4 | Hippocampus | tissue | |
| CAGE-seq | mouse | FANTOM5_phase1.3 | CNhs10474 | 5 | Lung | tissue | |
| CAGE-seq | mouse | FANTOM5_phase1.3 | CNhs10477 | 6 | Medulla oblongata | tissue | |
| CAGE-seq | mouse | FANTOM5_phase1.3 | CNhs10507 | 7 | Ovary | tissue | |
| CAGE-seq | mouse | FANTOM5_phase1.3 | CNhs10505 | 8 | Spinal cord | tissue | |
| CAGE-seq | mouse | FANTOM5_phase1.3 | CNhs10465 | 9 | Spleen | tissue | |
| CAGE-seq | mouse | FANTOM5_phase1.3 | CNhs10504 | 10 | Testis | tissue | |
| CAGE-seq | mouse | FANTOM5_phase1.3 | CNhs10613 | 11 | Universal | tissue | |
| TSS-seq | human | DBTSS_version_9 | NA | 1 | Adipose | tissue | ftp://ftp.hgc.jp/pub/hgc/db/dbtss/dbtss_ver9/hg38/TSSseq/tsc_data/Adult/ |
| TSS-seq | human | DBTSS_version_9 | NA | 2 | Adrenal | tissue | |
| TSS-seq | human | DBTSS_version_9 | NA | 3 | Brain (two repeats) | tissue | |
| TSS-seq | human | DBTSS_version_9 | NA | 4 | | tissue | |
| TSS-seq | human | DBTSS_version_9 | NA | 5 | Breast | tissue | |
| TSS-seq | human | DBTSS_version_9 | NA | 6 | Colon | tissue | |
| TSS-seq | human | DBTSS_version_9 | NA | 7 | Heart | tissue | |
| TSS-seq | human | DBTSS_version_9 | NA | 8 | Kidney | tissue | |
| TSS-seq | human | DBTSS_version_9 | NA | 9 | Liver | tissue | |
| TSS-seq | human | DBTSS_version_9 | NA | 10 | Lung | tissue | |

| | | | | | | | |
|---|---|---|---|---|---|---|---|
| TSS-seq | human | DBTSS_version_9 | NA | 11 | Lymph | tissue | |
| TSS-seq | human | DBTSS_version_9 | NA | 12 | Muscle | tissue | |
| TSS-seq | human | DBTSS_version_9 | NA | 13 | Ovary | tissue | |
| TSS-seq | human | DBTSS_version_9 | NA | 14 | Prostate | tissue | |
| TSS-seq | human | DBTSS_version_9 | NA | 15 | Testis | tissue | |
| TSS-seq | human | DBTSS_version_9 | NA | 16 | Thyroid | tissue | |
| TSS-seq | human | DBTSS_version_9 | NA | 1 | A427 | cell line | |
| TSS-seq | human | DBTSS_version_9 | NA | 2 | A549 | cell line | |
| TSS-seq | human | DBTSS_version_9 | NA | 3 | ABC1 | cell line | |
| TSS-seq | human | DBTSS_version_9 | NA | 4 | H1299 | cell line | |
| TSS-seq | human | DBTSS_version_9 | NA | 5 | H1437 | cell line | |
| TSS-seq | human | DBTSS_version_9 | NA | 6 | H1648 | cell line | |
| TSS-seq | human | DBTSS_version_9 | NA | 7 | H1650 | cell line | |
| TSS-seq | human | DBTSS_version_9 | NA | 8 | H1703 | cell line | |
| TSS-seq | human | DBTSS_version_9 | NA | 9 | H1819 | cell line | |
| TSS-seq | human | DBTSS_version_9 | NA | 10 | H1975 | cell line | |
| TSS-seq | human | DBTSS_version_9 | NA | 11 | H2126 | cell line | |
| TSS-seq | human | DBTSS_version_9 | NA | 12 | H2228 | cell line | |
| TSS-seq | human | DBTSS_version_9 | NA | 13 | H2347 | cell line | ftp://ftp.hgc.jp/pub/hgc/db/dbtss/dbtss_ver9/hg38/TSSseq/tsc_data/LA_CellLine_26/ |
| TSS-seq | human | DBTSS_version_9 | NA | 14 | H322 | cell line | |
| TSS-seq | human | DBTSS_version_9 | NA | 15 | II18 | cell line | |
| TSS-seq | human | DBTSS_version_9 | NA | 16 | LC2ad | cell line | |
| TSS-seq | human | DBTSS_version_9 | NA | 17 | PC14 | cell line | |
| TSS-seq | human | DBTSS_version_9 | NA | 18 | PC3 | cell line | |
| TSS-seq | human | DBTSS_version_9 | NA | 19 | PC7 | cell line | |
| TSS-seq | human | DBTSS_version_9 | NA | 20 | PC9 | cell line | |
| TSS-seq | human | DBTSS_version_9 | NA | 21 | RERFLCad1 | cell line | |
| TSS-seq | human | DBTSS_version_9 | NA | 22 | RERFLCad2 | cell line | |
| TSS-seq | human | DBTSS_version_9 | NA | 23 | RERFLCKJ | cell line | |
| TSS-seq | human | DBTSS_version_9 | NA | 24 | RERFLCMS | cell line | |
| TSS-seq | human | DBTSS_version_9 | NA | 25 | RERFLCOK | cell line | |
| TSS-seq | human | DBTSS_version_9 | NA | 26 | VMRCLCD | cell line | |
| GRO-cap | human | Core et al., 2014 | NA | NA | K562 | cell line | https://www.ncbi.nlm.nih.gov/geo/query/acc.cgi?acc=GSE60456 |
