# Supplementary material for: Evidence that alternative transcriptional initiation is largely nonadaptive
Source: PLoS Biol. 2019 Mar 18;17(3):e3000197. doi: 10.1371/journal.pbio.3000197 (PMC6438578; doi:10.1371/journal.pbio.3000197)
Supplement: S2 Table — TSS, transcription start site. (PDF) [file pbio.3000197.s013.pdf]

S2 Table. Correlations between gene expression level and TSS diversity or relative usage of a ranked TSS in simulated data.

Quantity being correlated with gene expression level	ρ (before down-sampling)	P (before down-sampling)	ρ (after down-sampling)	P (after down-sampling)
Simpson index of TSS diversity	0.0276	0.0011	0.0081	0.3388
Shannon index of TSS diversity	0.0859	3.3×10^{-24}	0.0065	0.4460
Relative usage of TSS of rank #1	-0.0165	0.0512	-0.0089	0.2920
Relative usage of TSS of rank #2	-0.0245	0.0046	0.0127	0.1429
Relative usage of TSS of rank #3	-0.0322	0.0003	0.0237	0.0080
Relative usage of TSS of rank #4	-0.0096	0.3026	0.0040	0.6659
